# Boron Triiodide-Mediated Reduction of Nitroarenes Using Borohydride Reagents

**DOI:** 10.1021/acs.orglett.3c03257

**Published:** 2023-12-05

**Authors:** Andrej Ćorković, Thomas Chiarella, Florence J. Williams

**Affiliations:** University of Iowa, Iowa City, Iowa 52242, United States

## Abstract

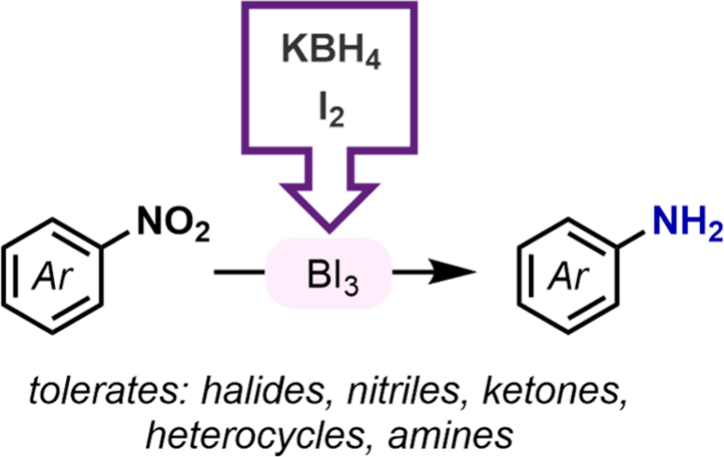

The reduction of nitroarenes using KBH_4_ and I_2_ is described. BI_3_ is generated in situ and was shown to be the active reductant. Conditions were optimized for BI_3_ generation and then applied to a wide range of nitroarenes, including traditionally challenging substrates. The method constitutes a practical reduction option which produces low-toxicity boric acid and potassium iodide upon workup.

The scale of demand is hard to comprehend for aniline derivatives. One of the most common aniline-containing medicinal compounds, acetaminophen (paracetamol), is generated on the scale of 180,000 tons yearly.^1^ In high-value medicinal and agrichemical structures, anilines are ubiquitous. Five of the top 10 small-molecule drugs in 2022 sales contain anilines (Eliquis, Revlimid, Trikafta, Xarelto, and Xtandi).

Due to the prevalence of this molecular unit within fine chemical scaffolds, it is important to continually revisit, diversify, and improve methods for its generation.^2^ Common industrial methods include (1) palladium-catalyzed hydrogenation of nitroarenes, (2) iron-mediated reduction of nitroarenes, and (3) conversion of phenols to anilines catalyzed through palladium-mediated redox chemistry.^3−5^ The third method is typically applied to unsubstituted aniline only;^6^ however, Li and co-workers have expanded the scope by utilizing hydrazine and LiOH.^7^

In 2015, Benaglia and co-workers described an elegant metal-free method to generate anilines using trichlorosilane and tertiary amines.^8^ Trichlorosilane is a cost-effective industrial byproduct. Further, the method boasts an impressive substrate scope, tolerating carboxylic acids, esters, and heterocycles, among others, and it will also reduce aliphatic nitro groups.

Transition-metal-free boron-mediated nitroarene reductions utilize diboron complexes (B_2_(OH)_4_, B_2_pin_2_, or B_2_nep_2_) and are another common choice for process and medicinal chemists. The byproducts have a modest toxicity profile (B_2_(OH)_4_ is an acute toxicity agent classified by OSHA as Category 4), and along with the Benaglia method, the substrate scope tends to be complementary to traditional metal-catalyzed strategies. For instance, aryl halides and nitriles are well-tolerated in these reductions, while such functional groups are regularly degraded in palladium-catalyzed hydrogenations. These methods require elevated temperature (≥100 °C),^10−13^ except for a method described by Han and co-workers, in which DMF solvent was observed to greatly accelerate the reaction at room temperature using B_2_(OH)_4_ and 4,4′-bipyridine.^14^

Herein we report a new method for the transition-metal-free reduction of nitroarenes using potassium borohydride and iodine (I_2_). This method satisfies many prerequisites for general adoption and diverse application: commercially available reagents, moderate reagent cost, and low toxicity of waste. Table 1 outlines commonly considered parameters for hazards, cost, and environmental impact under a few reduction conditions. The high cost of iodine is due to the 8 equivalents used and may also be related to costs associated with I_2_ being a regulated chemical. I_2_ is regenerated in the reaction (vide infra); therefore, there is potential for its recycling. Of note, our new method avoids flammable reagents and toxic waste since the key byproducts upon workup are boric acid and potassium iodide.

**Table 1:**
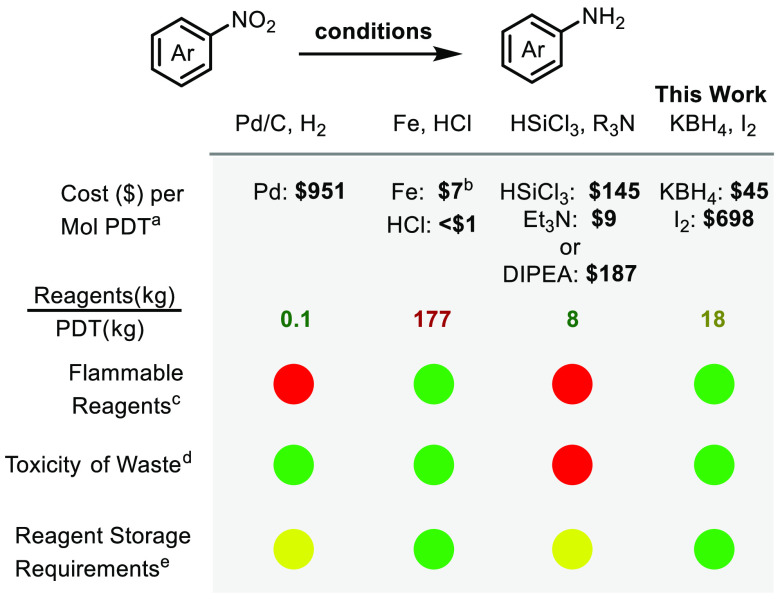
Common Nitroarene Reduction Methods^5,8,9^

a Cost per mol product (PDT) is calculated based on Sigma Aldrich catalogue costs on 9/20/2023 at largest (bulk) advertised rate, unless otherwise specified.

b Fe metal cost based on 9/20/2023 Fe scrap metal cost/ton.

c For flammability, red = OSHA Category <4 and green = no listed flammability hazard in SDS.

d For toxicity of waste, Red = acute toxicity (oral, dermal, or inhalation) OSHA Category <4; green = no listed acute toxicity (oral, dermal, or inhalation).

e Storage requirements refers to any of the following: pressurized gases, storage in glovebox, or requiring refrigeration. Yellow = one requirement; green = none.

Borohydride reagents have demonstrated utility in nitroarene reductions but to date have required transition metal catalysts and therefore constitute an alternative to hydrogen gas.^2^ However, these methods exhibit substrate scope limitations similar to those of other transition-metal-dependent reductions. An exception would be the use of iron(III) halides, as described by Thomas and colleagues,^15^ which has demonstrated utility with nitriles and aryl halides but modest yields with heterocyclic nitroarenes. To our delight, iodine functionally fills the role of a transition metal in our system. Importantly, borohydride itself does not appear to be the active reducing agent.

We first discovered the nitroarene reduction when examining the reactivity of a related reagent, boron triiodide (BI_3_). Although it is not a classical reductant, we observed the formation of aniline and the byproduct iodine when BI_3_ was added to nitroarenes (Scheme 1). We found that 2.5 equivalents of BI_3_ was optimal for this conversion.

**Scheme 1:**
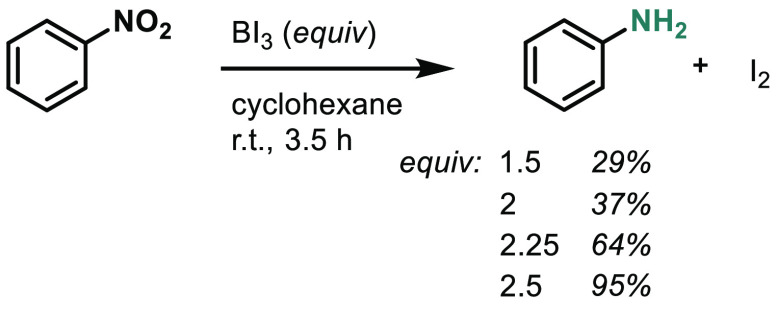
Reduction of Nitrobenzene with BI_3_

However, BI_3_ is an expensive reagent (∼$25,000/mol) that often needs to be purified by sublimation before use and stored in a glovebox. Thus, we sought methods to generate boron triiodide in situ and found a protocol outlined by Briggs and Simmons.^16^ We optimized the method further using ^11^B NMR to track BI_3_ production (Table 2; full optimization data are given in the Supporting Information (SI)).

**Table 2:**
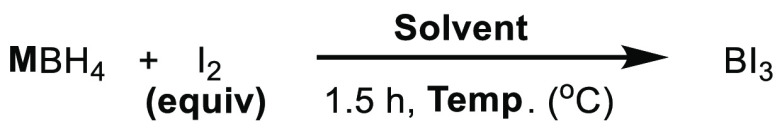
Optimization of BI_3_ Generation^a^

entry	M	mmol of MBH_4_	equiv of I_2_	temp. (°C)	solvent	mmol of BI_3_
1	K	0.50	2	60	cyclohexane	0.20^b^
2	K	0.50	2	60	cyclohexane	0.14^c^
3	K	0.50	2	60	cyclohexane	0.33
4	Li	0.50	2	60	cyclohexane	0.09
5	Na	0.54	2	60	cyclohexane	n.d.
6	K	0.50	1	60	cyclohexane	0.15
7	K	0.50	3	60	cyclohexane	n.d.
8	K	0.35	2	60	cyclohexane	0.26
9	K	0.35	2	70	heptane	0.27
10	K	0.35	2	60	DCM	n.d.

a All reactions were performed in a sealed 25 mL round-bottom flask with a rubber septum unless otherwise noted. n.d. = not detected.

b With a positive-pressure N_2_ gas inlet.

c With a positive-pressure N_2_ gas inlet and a purge needle open to air.

We discovered that a sealed vessel was critical for efficient BI_3_ generation (Table 2, entries 1–3). We hypothesize that the sealed vessel prevents the loss of HI as an intermediate in the conversion. Equations 1–4 outline our proposed balanced equation, requiring 2 equivalents of I_2_, which is supported by our optimal stoichiometry (entries 3, 6, and 7). It is unclear at this point why excess iodine results in no observable BI_3_ formation.

The production of H_2_ gas was confirmed by using a sample of the reaction atmosphere to reduce cyclooctadiene with palladium on carbon (see SI for details).
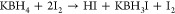
1
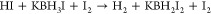
2
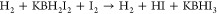
3
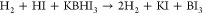
4

Potassium borohydride is uniquely suited to this transformation (Table 2, entries 3–5). While cyclohexane was selected for our optimization studies, we were gratified to see that heptane, a common industrial solvent, worked as well (entries 8–10).

Of note, hydrogen gas production and a modest pressure increase (estimated to be an additional ∼2 atm of pressure) do require engineering controls on scale. However, as BI_3_ production takes place prior to the addition of substrate, a single optimization protocol can be applied to a number of reactions. The subsequent nitroarene reductions were performed following removal of the produced H_2_ gas under standard pressure and room temperature.

We then turned our attention to the reduction of nitrobenzene. Pregeneration of BI_3_ by heating KBH_4_ and I_2_ is necessary, which provides evidence that BI_3_, rather than in situ-generated BH_3_, is the active reductant (Table 3, entry 1). Both KBH_4_ and I_2_ (entries 2 and 3) are required, and while some product is formed when all three are mixed and heated together (entry 4), the most efficient reaction occurs when BI_3_ is pregenerated (entry 5). Interestingly, 2-iodoaniline was consistently formed as a byproduct in about 5–10% NMR yield. This regioselectivity suggests possible iodine radicals, as radical additions to aromatic rings provide majority *ortho* substitution,^17^ in contrast to electrophilic aromatic substitution. Addition of alkenes as halide radical traps improved the reaction, with polystyrene polymers being optimal (entries 6–9). In our hands, Amberlite resins, which are polystyrene-based, were easiest to handle, as the polystyrene beads or powders form gelatinous semisolids in cyclohexane. The Amberlite resin could be reused four times without impact on the aniline yield.

**Table 3:**
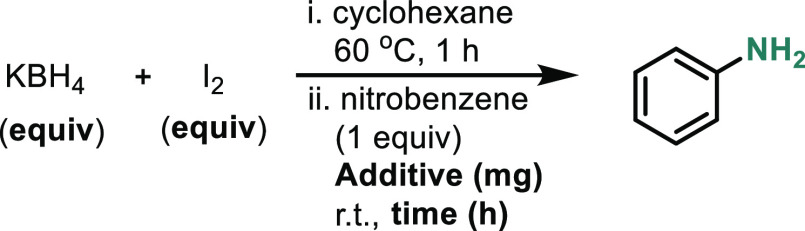
Optimization of Nitrobenzene Reduction^a^

entry	additive (mg)	equiv of KBH_4_	equiv of I_2_	time (h)	light?	NMR yield (%)
1^b^	none	10	10	1	Y	<5
2	none	5	0	1	Y	n.d
3	none	0	10	1	Y	n.d
4^c^	none	5	10	1	Y	58
5	none	5	10	1	Y	74
6	2-methyl-2-butene (56)	5	10	1	Y	78
7	polystyrene beads (220)	5	10	2	Y	84
8	polystyrene powder (190)	5	10	2	Y	94
9	Amberlite IR120 Na^+^ (200)	5	10	2	Y	94
10	Amberlite IR120 Na^+^ (100)	5	10	2	N	96
11	Amberlite IR120 Na^+^ (100)	3.5	7	2	Y	90
12	Amberlite IR120 Na^+^ (100)	4	8	1	Y	93

a All reactions were performed on a 0.2 mmol scale at 0.01 M. BI_3_ was generated in a 50 mL round-bottom flask. At the conclusion of the BI_3_ generation, the reaction mixture was purged with N_2_, then the nitroarene was added via syringe, followed by briefly exposing the reaction to air for additive addition, when necessary. Conversions are based on ^1^H NMR analysis with an internal standard.

b All reagents were added, without heating, at the same time instead of a two-step protocol.

c BI_3_ was not pregenerated. KBH_4_ and I_2_ were added with nitrobenzene and heated to 60 °C for 1 h.

Since BI_3_ is light-sensitive and had the potential for radical chemistry, we tested the reaction without light exposure and were surprised to find that it behaved identically in the dark as upon exposure to ambient light (Table 3, entries 9 and 10). We found that we could reduce the amount of BI_3_ used (entries 11 and 12), which corresponds well to our original BI_3_ optimal stoichiometry (Scheme 1). Of note, when the reaction was run on a 1 mmol scale, no exothermic response occurred upon addition of the substrate. This can be an important consideration in larger-scale applications (see SI section 1.6).

With the optimal reaction conditions in hand, we turned our attention to the substrate scope (Scheme 2). To our delight, several functional groups that are commonly problematic in nitroarene reductions performed well under our conditions, including cyano groups (**2e**), aryl halides (**2b**, **2c**, **2h**, **2i**), heterocycles (**2i**, **2j**, and **2m**), and ketones (**2f**). However, alkyl nitro groups (**1k**) did not provide the expected alkyl amine, and alkene **1n** was also problematic—both resulted in a complex mixture (**2k**, **2n**). For certain polar compounds, dichloromethane, dichloroethane, or trifluorotoluene was added to facilitate substrate solubility. Lewis basic groups, such as amine **2d** and nitrile **2e**, required additional equivalents of BI_3_ reagent, presumably to account for coordination and sequestration of the Lewis acidic reagent.

**Scheme 2:**
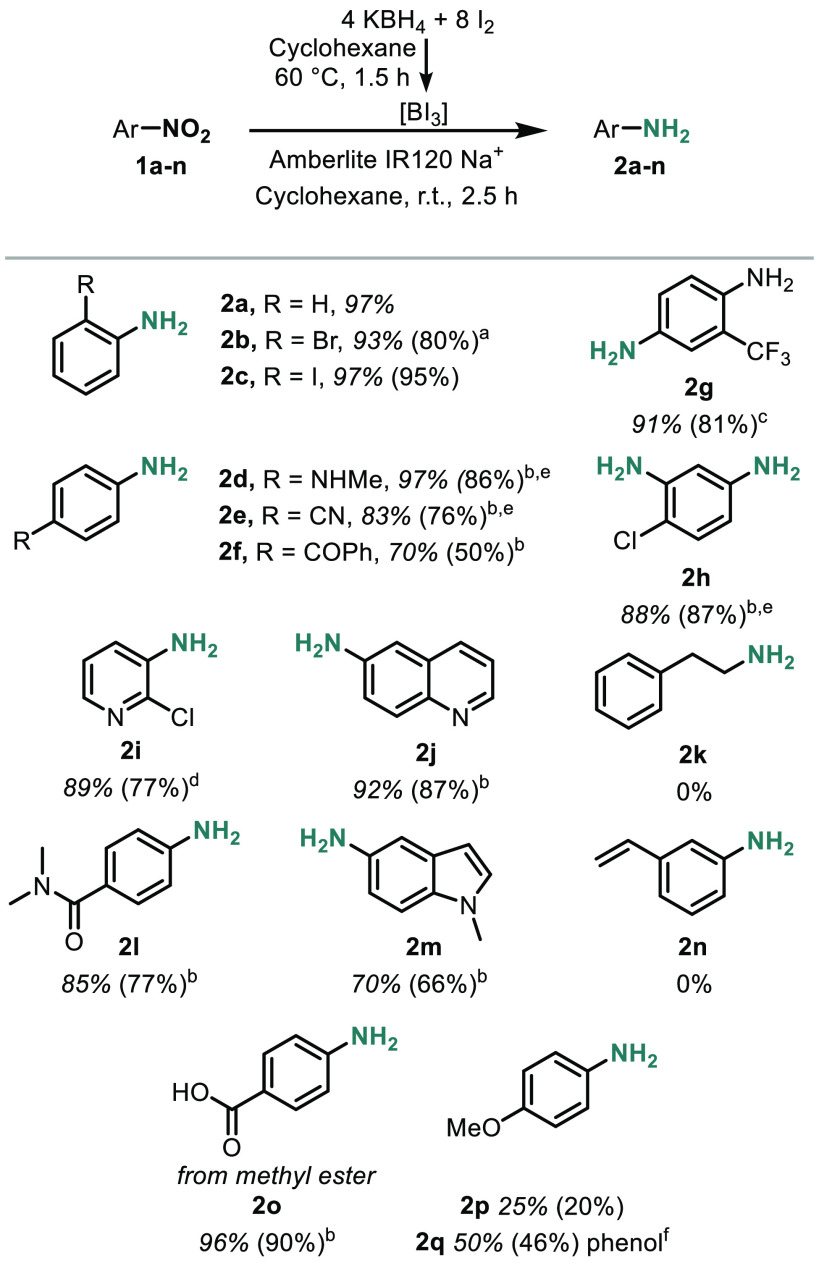
Substrate Scope All reactions were run on a 0.2 mmol scale at 0.01 M. BI_3_ was generated in a 50 mL round-bottom flask. At the conclusion of the BI_3_ generation, the reaction mixture was purged with N_2_, then the nitroarene was added via syringe, followed by briefly exposing the reaction to air for Amberlite resin addition. Conversions based on ^1^H NMR analysis with an internal standard are shown in italics. Isolated yields are shown in parentheses. Isolated as the HCl salt. 1,2-Dichloroethane was added as a cosolvent. Trifluorotoluene was added as a cosolvent. Dichloromethane was added as a cosolvent. Additional equivalents of KBH_4_ and I_2_ were used. Phenol **2q** was isolated from the reaction with **1p** along with **2p**.

Ester **1o** and ether **1p** reacted smoothly to afford the anilines but resulted in ester/ether cleavage to generate carboxylic acid/phenol products. While only carboxylic acid **2o** was recovered in the reaction with **1o**, nitroanisole substrate **1p** was converted to a mixture of *p*-aminophenol (**2q**) (50%) and *p*-anisidine (**2p**) (25%). Further optimization to maximize the yield of the ether may be feasible but was not attempted in this study.

It is notable that, in addition to ether **1p**, trifluoromethyl **1g** was fully tolerated, as trifluoromethyl and ether functional groups have known reactivity with related boron trihalide reagents (BBr_3_, BCl_3_).^18−22^

The tolerance of a ketone and amide coupled with no reduction of an ester are indicative of full or near-full consumption of the borohydride. No evidence of benzylic alcohol or benzylic amine was observed from these substrates.

Turning our attention to the mechanism of the reaction, we found no TEMPO adducts when TEMPO was used, and the reaction was only modestly affected (Scheme 3).

**Scheme 3:**
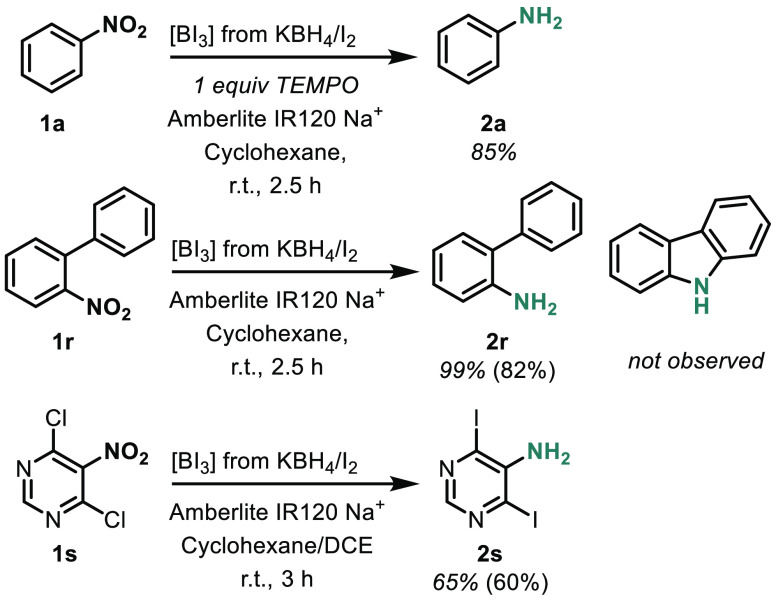
Mechanistic Test Substrates

We then took inspiration from previous diboron-mediated reactions. BI_3_ is known to form B_2_I_4_ and I_2_ under certain conditions,^23^ so it is not unreasonable to suspect that a diboron-mediated reaction could occur. Prior diboron-mediated mechanisms are proposed to involve nucleophilic boron species or nitrene/nitrenoid intermediates.^10,11,13^ We found the reaction conditions unlikely to support boron nucleophiles. We tested for nitrene/nitrenoid intermediates by using 2-nitrobiphenyl (**1r**) (Scheme 3). However, we did not observe carbazole and only observed the generation of 2-aminobiphenyl (**2r**). Another important consideration is whether HI is being produced and then causing the reduction. In our cyclohexane-solvated system, we expect only small amounts of HI to be generated. Since prior reports of HI-mediated nitroarene reduction were performed in 57% HI solutions at 90 °C^24^ and considering that the BI_3_ reaction proceeds at room temperature, we consider it unlikely that HI is the active reducing agent. Further, the reaction performs equally well in exhaustively dry cyclohexane.

Finally, it was observed that when electron-deficient heterocycle **1s** was used, reduction of the arene occurred alongside iodine–chlorine halogen exchange. This suggests that there is an iodide anion present in the reaction solution. Collectively, these mechanistic studies have led to a speculative mechanism outlined in the SI, involving nucleophilic attack of an iodide on a nitroarene-coordinated BI_3_, generating a new B–O bond, reducing the nitrogen center, and generating I_2_ as a byproduct.

BI_3_-mediated chemistry is underexplored in relation to its BCl_3_ and BBr_3_ relatives. This is likely due to its increased moisture and light sensitivity. Our protocol provides a straightforward method to generate BI_3_ from simple, inexpensive, and benchtop-stable reagents, which will be of broad benefit to the field of boron Lewis acid chemistry.

Herein we further describe nitroarene reductions using in situ-generated BI_3_. The practicality of the precursor reagents and the low toxicity of the byproducts provide compelling reasons for use in nitroarene reductions generally. The most expensive reagent, iodine, is also reproduced during the reduction process (a strong visual color change is observed that dissipates upon addition of thiol reductants), which provides an opportunity for recovery and reuse.

Despite the strong Lewis acidity of BI_3_, several functional groups which are often sensitive to reducing conditions (e.g., nitrile, ketone, halide) were well-tolerated and provided excellent conversion to the corresponding aniline. Further, functionality typically sensitive to boron Lewis acids, such as aryl alkyl ether and trifluoromethyl, were generally tolerated, save for some conversion of ether to the alcohol/phenol.

Mechanistically, the behavior of BI_3_ as a reductant is unexpected and may suggest additional opportunities for its utilization. We look forward to further exploring the chemical behavior of BI_3_ and its potential applications.

## Data Availability

All underlying data available in the article itself and its Supporting Information

## Supporting Information Available

Experimental procedures, characterization data, and NMR spectra for all compounds (PDF)
FAIR data, including the primary NMR FID files, for compounds **2b**–**2s** and BI_3_ in situ generation examples (ZIP)
