# Supplementary figures and images for: Boron Triiodide-Mediated Reduction of Nitroarenes Using Borohydride Reagents
Source: Org Lett. 2023 Dec 5;25(49):8787–91. doi: 10.1021/acs.orglett.3c03257 (PMC10729015; doi:10.1021/acs.orglett.3c03257)

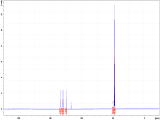

Supplement: Supplementary file 1 — ol3c03257_si_001.zip [file ol3c03257_si_001.zip › Nitro Reduction FIDs/Compound 2b 13C/2/pdata/1/thumb.png]

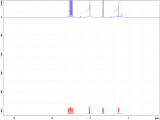

Supplement: Supplementary file 1 — ol3c03257_si_001.zip [file ol3c03257_si_001.zip › Nitro Reduction FIDs/Compound 2b 1H/3/pdata/1/thumb.png]

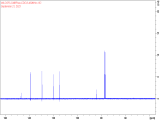

Supplement: Supplementary file 1 — ol3c03257_si_001.zip [file ol3c03257_si_001.zip › Nitro Reduction FIDs/Compound 2c 13C/1/pdata/1/thumb.png]

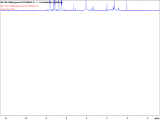

Supplement: Supplementary file 1 — ol3c03257_si_001.zip [file ol3c03257_si_001.zip › Nitro Reduction FIDs/Compound 2c 1H/1/pdata/1/thumb.png]

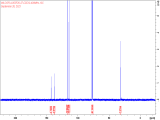

Supplement: Supplementary file 1 — ol3c03257_si_001.zip [file ol3c03257_si_001.zip › Nitro Reduction FIDs/Compound 2d 13C/1/pdata/1/thumb.png]

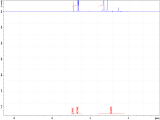

Supplement: Supplementary file 1 — ol3c03257_si_001.zip [file ol3c03257_si_001.zip › Nitro Reduction FIDs/Compound 2d 1H/1/pdata/1/thumb.png]

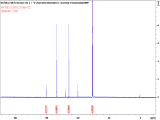

Supplement: Supplementary file 1 — ol3c03257_si_001.zip [file ol3c03257_si_001.zip › Nitro Reduction FIDs/Compound 2e 13C/2/pdata/1/thumb.png]
