# Supplementary material for: Boron Triiodide-Mediated Reduction of Nitroarenes Using Borohydride Reagents
Source: Org Lett. 2023 Dec 5;25(49):8787–91. doi: 10.1021/acs.orglett.3c03257 (PMC10729015; doi:10.1021/acs.orglett.3c03257)
Supplement: Supplementary file 2 — ol3c03257_si_002.pdf [file ol3c03257_si_002.pdf]

Supporting Information

Boron Triiodide-Mediated Reduction of Nitroarenes Using Borohydride Reagents

Andrej Ćorković,[a] Thomas Chiarella,[a] and Florence J. Williams *[a]
[a]University of Iowa, Iowa City, IA, 52242

Email: florence-williams@uiowa.edu

1.1. General Information S-2
1.2. Synthesis of Aniline Products S-3
1.3. Optimization of BI_3 Generation S-4
1.4. Optimization of Nitrobenzene Reduction S-6
1.5. Miscellaneous Reactions S-8
1.6. 1 mmol Scale Reaction S-8
1.7. Speculative Mechanistic Proposal S-9
1.8. Characterization of Amine Products S-10
1.9. Spectral Data S-14

2. References S-31

1. Supplemental Data

1.1. General Information

Deuterated methanol (CD_3OD) and deuterated chloroform ($CDCl_3$) were stored in a container with oven-dried (>200 °C) molecular sieves (4 Å). RPM = revolutions per minute, DCE = 1,2-dichloroethane, DCM = dichloromethane, TFT = α,α,α-trifluorotoluene, EtOAc = ethyl acetate, Hex = hexanes, TEA = triethylamine, DTT = dithiothreitol, MeOH = methanol, ACN = acetonitrile, TFA = trifluoroacetic acid. Thin-layer chromatography was performed on glass-plates pre-coated with 0.25 mm silica gel with a fluoresecent indicator UV_{254} (EMD Millipore). Chromatography columns were packed with 230-400 mesh silica gel (Sigma-Aldrich). HPLC (High Performance Liquid Chromatography) purification was performed on an Agilent 1260 instrument with a 1290 pump using an Agilent 10 Prep C18 column (150 mm x 22 mm, 10μm particle size). GCMS (Gas Chromatography Mass Spectrometry) was performed on a Waters GCT Premier. All reagents were purchased from Sigma Aldrich, ThermoFisher Scientific, TCI, Alfa Aesar, AmBeed, Matrix, Acros Organics, and Cambridge Isotope Laboratories, Inc. with purity ≥97%. Hydrogen nuclear magnetic resonance (^{1}H-NMR) spectra were recorded at 300, 400, or 500 MHz Bruker instruments, and coupling constants (J) are reported in Hertz (Hz). Internal standards used for ^{1}H-NMR are described for each substrate below. Carbon nuclear magnetic resonance (^{13}C-NMR) spectra were recorded at 100, 125, or 150 MHz. Boron nuclear magnetic resonance (^{11}B-NMR) spectra were recorded at 96 or 128 MHz, using a quartz NMR tube. Boron concentrations were calculated using a $BF_3{\cdot}OEt_2$ capillary internal standard. Fluorine nuclear magnetic resonance (^{19}F-NMR) spectra were recorded at 282 MHz. Chemical shifts are reported in δ (ppm) and referenced to the residual protonated solvent (^{1}H) peaks, to the deuterated solvent (^{13}C) peaks, or to the $BF_3{\cdot}OEt_2$ internal standard peaks: $CDCl_3$ (7.26 ppm, ^{1}H; 77.16 ppm, ^{13}C), CD_3OD (3.31 ppm, ^{1}H; 49.00 ppm, ^{13}C) and $BF_3{\cdot}OEt_2$ (0 ppm, ^{11}B). s = singlet, d = doublet, t = triplet, q = quartet, bs = broad singlet, m = multiplet, app. = apparent, etc. The $BF_3{\cdot}OEt_2$ capillary internal standard was prepared by sealing a $BF_3{\cdot}OEt_2$:$CDCl_3$ solution into a capillary. The internal standard integration value was calibrated by comparing to a known solution of phenylboronic acid (60. mg, 0.49 mmol) in tetrahydrofuran (THF) (5.0 mL, 0.10 M) through ^{11}B-NMR. Mp = melting point.

Safety Note: *The generation of BI_3 occurs in a sealed heated vessel and produces flammable H_2 gas as a byproduct. As such, it should be done behind a blast shield on small scale and with proper engineering controls on larger scales to mitigate risks of exotherm, explosion, or fire. Further, attention should be paid to the size of the reaction vessel relative to the H_2 gas (and increased pressure) produced.*

Synthesis of Starting Reagents

Synthesis of ***1l****:*

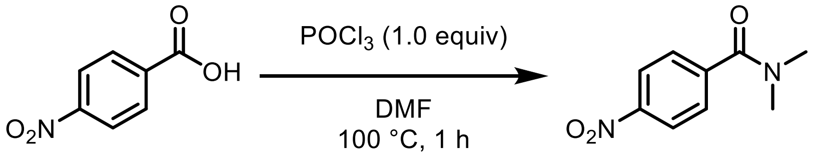

1l

Synthesis of **1l** was carried out following literature procedure.[1]

*Synthesis of **1m**:*

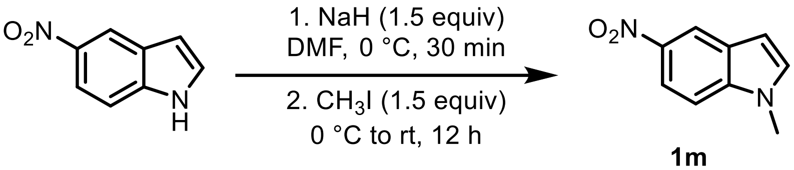

1m

Synthesis of **1m** was carried out following literature procedure.[2]

*Synthesis of **1o**:*

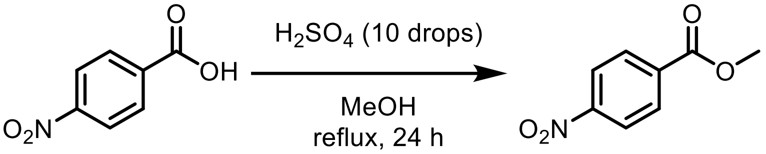

1o

Synthesis of **1o** was carried out following literature procedure.[3]

1.2. Synthesis of Aniline Products

General Procedure: Reduction of Nitro Compounds via in situ Generated Boron Triiodide (BI_3)

Potassium borohydride (KBH_4) (43.2 mg, 0.800 mmol, 4.00 equiv) was added to a clean 50 mL RBF and ground with a glass stir rod for 30-60 s. Molecular iodine (I_2) (406.1 mg, 1.600 mmol, 8.000 equiv), cyclohexane (4 mL), and an oven-dried (80 °C) stir bar were subsequently added. The flask was sealed with a rubber septum secured by copper wire. The mixture was heated to 60 °C in a silicon oil bath and stirred at 700 RPM for 1.5 h. The reaction flask was allowed to cool to room temperature (pressure buildup of byproduct gases observed with inflated septum). The septum was simultaneously pierced with two 20-gauge needles, one to evacuate gaseous byproducts and the other to introduce a nitrogen atmosphere. Completed synthesis of BI_3 was usually accompanied with the loss of purple color attributed to iodine. The starting nitro compound (0.20 mmol, 1.0 equiv) was dissolved in cyclohexane (17 mL). This solution was added to the reaction flask containing in situ generated BI_3. The septum was quickly removed, and Amberlite ™ IR120 Na+ form (100. mg) was added before quickly recapping the reaction flask with the septum. Nitrogen was introduced to the reaction flask, and the mixture was stirred at room temperature for 2.5 h.

*Note – for substrates **2d-j**, **2l-m**, **2o**, and **2s** a cosolvent (trifluorotoluene, DCM, or DCE) was used with cyclohexane. Please see Characterization Data section **1.8** for the identity and solvent ratio used for these substrates.

Workup 1: The mixture was cooled to 0 °C and MeOH (~5.0 mL) was added. DTT (~50 mg) was added to react with excess iodine (A loss of color was accompanied with the addition of the reducing agent. If significant color remained, additional DTT was added until color receded). TEA (~1.0 mL) was added to the mixture, which was then vacuum filtered and concentrated in vacuo.

Workup 2: The mixture was cooled to 0 °C and sat. aq. $Na_2S_2O_3$ (~10 mL) was added with stirring. The resultant biphasic cloudy solution was added to a separatory funnel, and shaken until both solutions became colorless, or until the organic layer color was observed not to change upon further added sat. aq. $Na_2S_2O_3$. The aqueous layer was separated and neutralized with $NaHCO_3$ (solid or sat. aq. solution). Once neutralized,

the aqueous layer was added back into the separatory funnel and extracted with EtOAc (3x20 mL), DCM (3x20 mL), or ether (3x20 mL). If cloudy, the organic layers were washed with sat. brine (10 mL), then dried over Na_2SO_4 and concentrated in vacuo.

1,3-benzodioxole or 1,3,5-trimethoxybenzene were added directly to the crude residue as internal standards. The crude residue and internal standard were dissolved in CD_3OD or $CDCl_3$. NMR analysis proceeded with quantification of amino products determined relative to internal standard. The residue was purified by column chromatography (EtOAc:Hex:TEA, MeOH in DCM, pentane:ether) on silica gel to afford the desired product.

*Note – DTT was utilized to facilitate internal standard-based conversions without concern for sequestration of the product(s) into aqueous layers. However, the use of a sat. aq. $Na_2S_2O_3$ wash to remove excess iodine was also demonstrated to be effective as a part of the reaction workup, which avoids separation of DTT from the reaction product(s).

1.3. Optimization of BI_3 Generation

In situ Synthesis of BI_3 Optimization

A borohydride (0.50 mmol or 0.35 mmol, 1.0 equiv) was added to a clean 25 mL RBF and ground with a glass stir rod for 30-60 s. I_2, at the described equivalents, solvent, and an oven-dried (80 °C) stir bar were subsequently added before capping the flask with a rubber septum. The mixture was heated to the desired temperature in a silicon oil bath and stirred for 1.5 h. The reaction flask was cooled to room temperature (pressure buildup of byproduct gases observed with inflated septum). At the conclusion of the reaction, the septum was simultaneously pierced with two 20-gauge needles, one to evacuate gaseous byproducts and the other to introduce a nitrogen atmosphere. Completed synthesis of BI_3 was usually accompanied with the loss of purple color attributed to iodine (Figure S1). The reaction mixture was transferred to a quartz NMR tube equipped with the sealed capillary containing $BF_3{\cdot}OEt_2$. Analysis with ^{11}B-NMR identified BI_3 (-8.00 ppm), and any boron-containing byproducts which were quantified relative to the $BF_3{\cdot}OEt_2$ internal standard (0.00 ppm) (Figure S2) (Table S1).

Figure S1. Loss of color attributed to consumption of iodine.

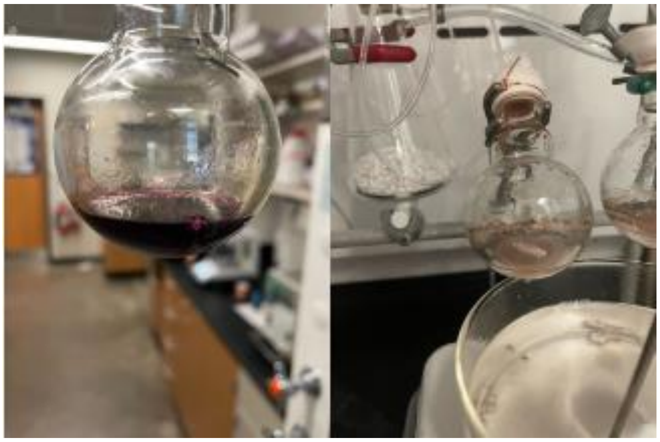

Left: Mixture of KBH_4, I_2, and cyclohexane before heating. Right: Mixture of BI_3, salts, and cyclohexane after heating.

Figure S2. Examples of BI_3 generation ^{11}B-NMRs with internal standard.

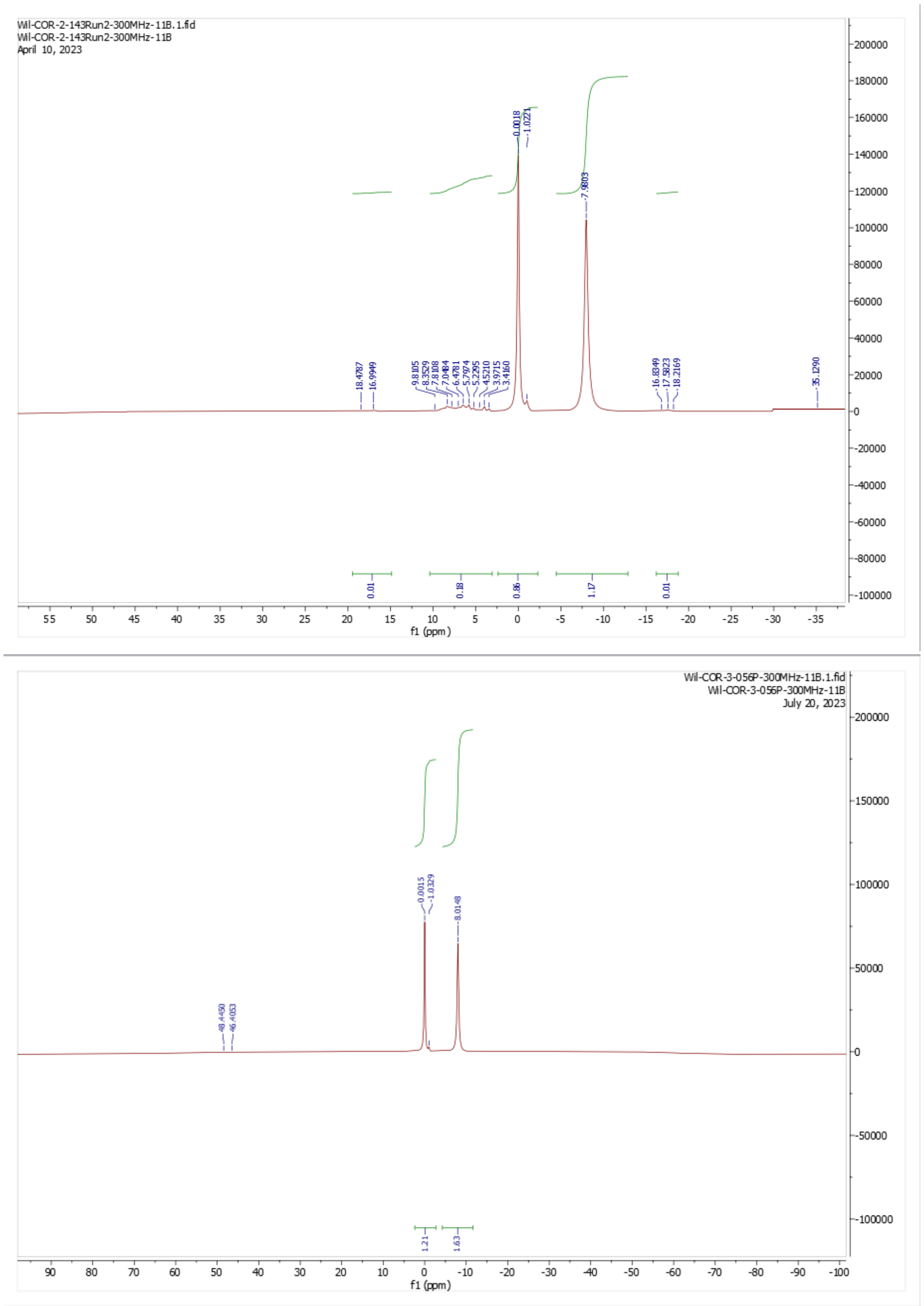

(Top) An example of a BI_3 generation reaction which was inefficient. (Bottom) An example of a BI_3 generation reaction which was efficient and deemed suitable for subsequent use.

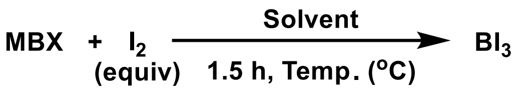

Table S1. Optimization of BI_3 generation

Entry	M	X	MBX (mmol)	I_2 (equiv)	Temp (°C)	Solvent	Solvent Volume (mL)	Light ?	BI_3 (mmol)
1	K	BH_4	0.50	2	60	Cyclohexane	2	Yes	0.20[a]
2	K	BH_4	0.50	2	60	Cyclohexane	2	Yes	0.14[b]
3	K	BH_4	0.50	2	60	Cyclohexane	2	Yes	0.33
4	Li	BH_4	0.35	2	60	Cyclohexane	2	Yes	0.05
5	Li	BH_4	0.50	2	60	Cyclohexane	2	Yes	0.09
6	Na	BH_4	0.54	2	60	Cyclohexane	2	Yes	n. d.
7	K	BH_4	0.50	1	60	Cyclohexane	2	Yes	0.15
8	K	BH_4	0.50	3	60	Cyclohexane	2	Yes	n. d.
9	K	BH_4	0.35	2	60	Cyclohexane	2	Yes	0.26
10	K	BH_4	0.35	2	70	Heptane	2	Yes	0.27
11	K	BH_4	0.35	2	60	DCM	2	Yes	n. d.
12	Na	BH_3CN	0.50	2	60	Cyclohexane	2	Yes	n. d.
13	K	BH_4	0.35	2	60	Heptane	2	Yes	0.16
14	K	BH_4	0.35	2	60	Toluene	2	Yes	n. d.
15	K	BH_4	0.35	2	60	Cyclohexane	2	No	0.23
16	K	BH_4	0.50	2	60	Cyclohexane	1	Yes	0.22
17	K	BH_4	0.50	2	60	Cyclohexane	3	Yes	0.30
18	K	BH_4	0.50	2	60	Cyclohexane	5	Yes	0.30

n. d. = not detected. [a]with positive pressure N_2 gas inlet. [b]with positive pressure N_2 gas inlet and a purge needle open to air.

1.4. Optimization of Nitrobenzene Reduction

Reduction of Nitro Compounds Optimization

Nitrobenzene (12 mg, 0.10 mmol, 1.0 equiv) was dissolved in a target volume of cyclohexane. This solution was transferred to the reaction flask containing in situ generated BI_3. This mixture was stirred at a target temperature for the listed amount of time. The reaction was quenched, and a sulfur-based reducing agent

was added to remove excess I_2 (A loss of color was accompanied with the addition of the reducing agent). The contents of the reaction flask were concentrated in vacuo. Internal standard 1,3-benzodioxole was added directly to the crude residue prior to obtaining NMR spectra. Conversion to the aniline was determined by ^{1}H-NMR using 1,3-benzodioxole internal standard (Table S2).

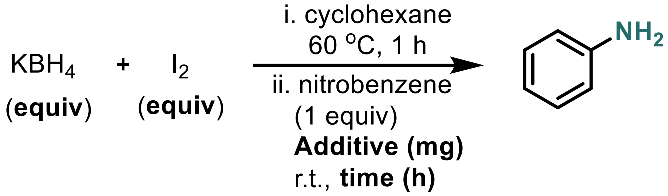

Table S2: Optimization of Nitrobenzene Reduction

Entry	Additive	Amt (mg)	KBH_4 (equiv)	I_2 (equiv)	Time (h)	Light?	Concentration (M)	NMR Yield
1	None	n/a	5	0	1	Yes	0.01	n. d.
2	None	n/a	0	10	1	Yes	0.01	n. d.
3	None	n/a	5	10	1	Yes	0.01	58[a]
4	None	n/a	5	10	1	Yes	0.01	74
5	2-methyl-2-butene	56	5	10	1	Yes	0.01	78
6	Polystyrene beads	220	5	10	2	Yes	0.01	84
7	Polystyrene powder	190	5	10	2	Yes	0.01	94
8	Amberlite IR120 Na^+	200	5	10	2	Yes	0.01	94
9	Amberlite IR120 Na^+	100	5	10	2	No	0.01	96
10	Amberlite IR120 Na^+	100	3.5	7	2	No	0.01	93
11	Amberlite IR120 Na^+	100	3.5	7	2.5	Yes	0.01	90
12	Amberlite IR120 Na^+	100	4	8	1	Yes	0.01	93
13	Amberlite IR120H ion-exchange resin	200	5	10	2	Yes	0.01	88
14	N,N'-dimethylaniline	17	5	10	1	Yes	0.03	81
15	p-toluenethiol	26	5	10	1	Yes	0.03	60
16	1,5-cyclooctadiene	14	5	10	1	Yes	0.03	71
17	TFT	82	5	10	1	Yes	0.03	74
18	DTT	10	5	10	2	Yes	0.01	85

[a]BI_3 was not pre-generated. KBH_4 and I_2 were added with nitrobenzene and heated to 60 °C for 1 h.

1.5. Miscellaneous Reactions

To determine the contents of the flask containing the in situ generated BI_3, several hydrogenation reactions were performed:

Palladium on carbon (76.7 mg, 0.720 mmol, 10 mol%) was added to a dried 25 mL RBF. Ethanol (6 mL) and 1,5-cyclooctadiene (24.5 µL, 0.200 mmol, 1 equiv) were subsequently added. To the sealed flask, 10 mL of headspace atmosphere were removed via syringe to generate a temporary vacuum. Subsequently, 10 mL of headspace atmosphere from an in situ BI_3 generation reaction was added (BI_3 was generated using heating and sonication). The reaction was allowed to stir for 1.5 h. At the conclusion of the reaction, a positive pressure nitrogen gas inlet was added as well as a needle to the external atmosphere to flush with N_2 gas. The reaction contents were filtered through celite, and the filtrate was washed with ethanol. The sample was subsequently submitted for GCMS analysis.

Additionally, a negative control was conducted following the same procedure but without the presence of palladium on carbon.

1.6. 1 mmol Scale Reaction

KBH_4 (215.8 mg, 4.000 mmol, 4.000 equiv) was added to a clean single-neck 250 mL RBF and ground with a glass stir rod for 30-60 s. I_2 (2.03 g, 8.00 mmol, 8.00 equiv), cyclohexane (20 mL), and an oven-dried (80 °C) stir bar were subsequently added. The flask was sealed with a rubber septum secured by copper wire. The mixture was heated to 60 °C in a silicon oil bath and stirred at 700 RPM for 1.5 h. The reaction flask was allowed to cool to room temperature and pierced with one 20-gauge needle with positive pressure N_2 and one 20-gauge needle open to air to evacuate gaseous byproducts. This was allowed to purge for 10 minutes prior the removal of the needle open to air. 1-iodo-2-nitrobenzene (249.0 mg, 1.000 mmol, 1.000 equiv) was dissolved in cyclohexane (85 mL) and injected into the reaction flask containing in situ generated BI_3 over approximately 2 min. The septum was quickly removed, and Amberlite ™ IR120 Na+ form (500. mg) was added before quickly recapping the reaction flask with the septum. Nitrogen was introduced to the reaction flask, and the mixture was stirred at room temperature for 2.5 h.

After 2.5 h, the mixture was cooled to 0 °C and sat. aq. $Na_2S_2O_3$ (50 mL) was added with stirring. The resultant biphasic cloudy solution was added to a separatory funnel and shaken until both solutions became colorless. The aqueous layer was separated and neutralized with $NaHCO_3$ (~0.5 g solid and ~10 mL sat. aq. solution). Minor, but not vigorous, bubbling from CO_2 production was observed. The aqueous layer was added to the separatory funnel and extracted with EtOAc (2x40 mL) and DCM (2x40 mL). The organic layers were combined, dried with Na_2SO_4, and concentrated in vacuo. Conversion to 2-iodoaniline (94%) was determined by ^{1}H-NMR using 1,3-benzodioxole as an internal standard.

The compound was purified by column chromatography (95:4:1 to 90:9:1 Hex:EtOAc:TEA) to obtain 171 mg (78% yield) of the desired 2-iodoaniline as a white solid. mp: 54-56 °C. R_f = 0.35 with 90:10 Hex:EtOAc. Spectral data were consistent with compound 2c.

This procedure was repeated with a 3-neck 250 mL RBF fitted with an internal thermometer and two septa to measure any temperature changes upon addition of the 1-iodo-2-nitrobenzene substrate. The temperature read 25.0 °C prior to the addition of the substrate, and 25.5 °C following addition of the substrate. The conversion to the aniline at the conclusion of the reaction was 70% by ^{1}H-NMR with 1,3-benzodioxole as an internal standard.

1.7. Speculative Mechanistic Proposal

Based on our observations, we propose the speculative mechanism outlined in Scheme S1. Iodide anion could react with a coordinated BI_3 group in **A**, creating a B–O bond and reducing the nitrogen center. Upon formation of intermediate **C**, another equivalent of iodide could react with BI_3 generating BI_2O and intermediate **D**. A repeat of this sequence would produce product **F**, which hydrolyses to aniline upon workup. The BI_2O byproduct would quickly form boroxine **G** and iodide in solution. Iodine radicals, if formed, could be produced from coordination of BI_3 with the nitroarene if the B–I bond (66.5-68 kcal/mol in BI_3) is sufficiently weakened to homolyze.[4,5] Cyclic diboron complex **C**, could go through a similar mechanistic sequence to reduce the nitroarene.

Several alternative mechanistic possibilities remain, and we intend to further interrogate the mechanism in subsequent work.

Scheme S1. Mechanistic Proposal.

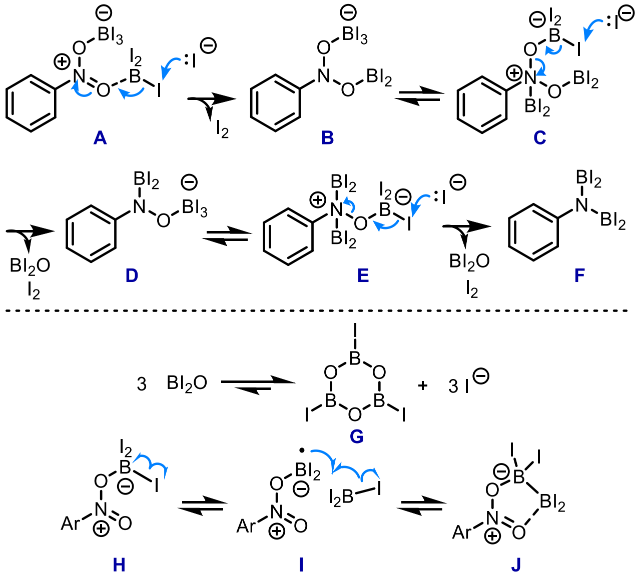

1.8. Characterization of Amine Products

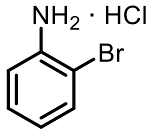

2-bromoaniline hydrochloride (2b)

33.4 mg (80%) was isolated as a white solid following the general procedure and workup 2 with EtOAc, followed by the addition of 1 mL conc. HCl. ^{1}H-NMR (CD_3OD, 400 MHz): δ 7.79 (dd, J = 8.0, 1.3 Hz, 1H), 7.59 (dd, J = 8.0, 1.6 Hz, 1H), 7.53 (td, J = 7.9, 1.3 Hz, 1H), 7.41 (td, J = 7.9, 1.7 Hz, 1H) ppm. ^{13}C-NMR (CD_3OD, 100 MHz): δ 135.1, 131.7, 130.4, 125.9, 118.1 ppm. No further purification was required following workup. R_f = 0.25 with 100% EtOAc. Compound became a black tar at 200 °C upon obtaining the melting point (mp). Spectral data were consistent with literature reports.[6]

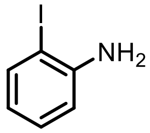

2-iodoaniline (2c)

41.9 mg (95%) was isolated as a white solid following the general procedure and workup 1. ^{1}H-NMR ($CDCl_3$, 300 MHz): δ 7.64 (dd, J = 7.9, 1.4 Hz, 1H), 7.14 (td, J = 7.8, 1.4 Hz, 1H), 6.75 (dd, J = 8.0, 1.5 Hz, 1H), 6.48 (td, J = 7.8, 1.5 Hz, 1H), 4.08 (bs, 2H) ppm. ^{13}C-NMR ($CDCl_3$, 75 MHz): δ 146.9, 139.1, 129.5, 120.1, 114.9, 84.3 ppm. Purified by column chromatography (95:4:1 Hex:EtOAc:TEA), R_f = 0.29. mp: 53-55 °C (lit. 55-58 °C). Spectral data were consistent with literature reports.[7]

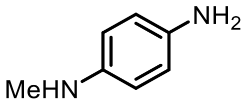

4-amino-N-methylaniline (2d)

22.0 mg (86%) was isolated as a brown solid following a modified procedure and workup 2 with EtOAc. A 2:3 mixture of DCE and cyclohexane was used as the solvent in the nitro reduction. Additionally, 6 equivalents of KBH_4 and 12 equivalents of I_2 were used. ^{1}H-NMR ($CDCl_3$, 400 MHz): δ 6.63 (d, J = 8.6 Hz, 2H), 6.52 (dd, J = 8.6, 2.1 Hz, 2H), 3.22 (bs, 3H), 2.79 (s, 3H) ppm. ^{13}C-NMR ($CDCl_3$, 100 MHz): δ 142.8, 137.8, 117.0, 114.2, 31.9 ppm. Purified by column chromatography (60:39:1 EtOAc:Hex:TEA), R_f = 0.25. A melting point was not obtained for this compound because it was observed to oxidize within hours of purification. Spectral data were consistent with literature reports.[8]

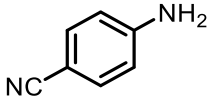

4-aminobenzonitrile (2e)

18.0 mg (76%) was isolated as a pale yellow solid following a modified procedure and workup 1. A 3:17 mixture of DCE and cyclohexane was used as the solvent in the nitro reduction. Additionally, 6 equivalents of KBH_4 and 12 equivalents of I_2 were used. ^{1}H-NMR ($CDCl_3$, 400 MHz): δ 7.40 (d, J = 8.7 Hz, 2H), 6.64 (d, J = 8.8 Hz, 2H), 4.16 (bs, 2H) ppm. ^{13}C-NMR ($CDCl_3$, 100 MHz): δ 150.5, 133.9, 120.2, 114.5, 100.2 ppm. Purified by column chromatography (65:34:1 Hex:EtAOc:TEA), R_f = 0.17 with 70:30 Hex:EtOAc. mp: 81-83 °C (lit. 76-81 °C). Spectral data were consistent with literature reports.[9]

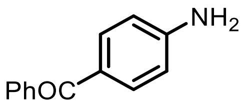 *4-aminobenzophenone (2f)*

19.5 mg (50%) was isolated as a yellow solid following a modified procedure and workup 1. A 3:17 mixture of DCE and cyclohexane was used as the solvent in the nitro reduction. ^{1}H-NMR ($CDCl_3$, 400 MHz): δ 7.72-7.69 (m, 4H), 7.52 (tt, J = 7.3, 1.3 Hz, 1H), 7.44 (t, J = 7.6 Hz, 2H), 6.65 (d, J = 8.7 Hz, 2H), 4.17 (bs, 2H) ppm. ^{13}C-NMR ($CDCl_3$, 100 MHz): δ 195.5, 151.1, 139.0, 133.1, 131.5, 129.6, 128.2, 127.5, 113.7 ppm. Purified by column chromatography (75:24:1 Hex:EtOAc:TEA), R_f = 0.18 with 70:30 Hex:EtOAc. mp: 121-122 °C (lit. 122-123 °C). Spectral data were consistent with literature reports.[10]

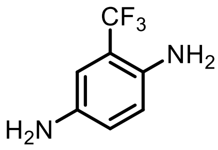 *2-(Trifluoromethyl)-1,4-phenylenediamine (2g)*

28.4 mg (81%) was isolated as a brown solid following a modified procedure and workup 2 with DCM. A 3:17 mixture of TFT and cyclohexane was used as the solvent in the nitro reduction. ^{1}H-NMR (400 MHz, $CDCl_3$): δ 6.81 (d, J = 2.7 Hz, 1H), 6.71 (app. ddd, J = 8.5, 2.6, 0.5 Hz, 1H), 6.61 (d, J = 8.5 Hz, 1H), 3.61 (bs, 4H) ppm. ^{13}C-NMR (100 MHz, $CDCl_3$): δ 138.0, 136.8 (q, J = 1.9 Hz), 124.9 (q, J = 270.6 Hz), 120.8, 119.2, 115.3 (q, J = 29.6 Hz), 113.1 (q, J = 5.3 Hz) ppm. ^{19}F-NMR (282 MHz, $CDCl_3$): δ -62.7 (s, 3F) ppm. ^{19}F-NMR was calibrated using 4-fluorotoluene as an internal standard at -118.82 ppm.[11] Purified by column chromatography (50:50 pentane:ether), R_f = 0.15 with 70:30 Hex:EtOAc. mp: 51-53 °C (lit. 55-57 °C). Spectral data were consistent with literature reports.[12,13]

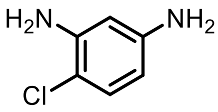 *2-chloro-1,4-diaminobenzene (2h)*

24.3 mg (87%) was isolated as a white solid following a modified procedure and workup 2 with EtOAc. A 1:3 mixture of DCE and cyclohexane was used as the solvent in the nitro reduction. Additionally, 8 equivalents of KBH_4 and 16 equivalents of I_2 were used. ^{1}H-NMR ($CDCl_3$, 400 MHz): δ 6.99 (d, J = 8.4 Hz, 1H), 6.09 (d, J = 2.5 Hz, 1H), 6.06 (dd, J = 8.4, 2.5 Hz, 1H), 3.90 (bs, 2H), 3.58 (bs, 2H) ppm. ^{13}C-NMR ($CDCl_3$, 100 MHz): δ 146.3, 143.5, 130.0, 109.4, 106.8, 102.4 ppm. No further purification was required following workup. mp: 83-85 °C (lit. 71-73 °C). Spectral data were consistent with literature reports.[14]

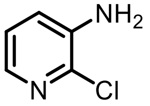 *3-amino-2-chloropyridine (2i)*

19.9 mg (77%) was isolated as a tan solid following a modified procedure and workup 1. A 1:1 mixture of DCM and cyclohexane was used as the solvent. ^{1}H-NMR ($CDCl_3$, 300 MHz): δ 7.79 (t, J = 3.0 Hz, 1H), 7.03 (d, J = 3.3 Hz, 2H), 4.10 (bs, 2H) ppm. ^{13}C-NMR ($CDCl_3$, 100 MHz): δ 139.8, 138.8, 137.1, 123.5, 122.5 ppm. Purified by column chromatography (80:19:1 Hex:EtOAc:TEA), R_f = 0.19. mp: 74-77 °C (lit. 77-79 °C). Spectral data were consistent with literature reports.[9]

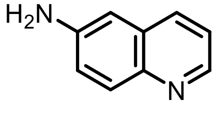

6-aminoquinoline (2j)

25.0 mg (87%) was isolated as an off-white solid following a modified procedure and workup 2 with DCM. A 3:17 mixture of DCE and cyclohexane was used as the solvent. ^{1}H-NMR ($CDCl_3$, 400 MHz): δ 8.64 (dd, J = 4.2, 1.6 Hz, 1H), 7.91-7.86 (m, 2H), 7.24 (dd, J = 8.2, 4.2 Hz, 1H), 7.13 (dd, J = 8.9, 2.6 Hz, 1H), 6.87 (d, J = 2.6 Hz, 1H), 3.86 (bs, 2H) ppm. ^{13}C-NMR ($CDCl_3$, 100 MHz): δ 146.9, 144.7, 143.5, 133.9, 130.6, 129.9, 121.7, 121.5, 107.5 ppm. Purified by column chromatography (90:10 ether:pentane to 100% ether), R_f = 0.30 with 95:5 DCM:MeOH. mp: 115-117 °C (lit. 113-116 °C). Spectral data were consistent with literature reports.[9] Contains 3% BHT (butylated hydroxytoluene) from the ether used in purification in the spectra.

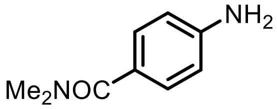

4-amino-N,N-dimethylbenzamide (2l)

25.4 mg (77%) was isolated as a white solid following the general procedure and workup 2 with both EtOAc and DCM. ^{1}H-NMR ($CDCl_3$, 400 MHz): δ 7.20 (d, J = 8.6 Hz, 2H), 6.56 (d, J = 8.6 Hz, 2H), 3.84 (bs, 2H), 2.97 (s, 6H) ppm. ^{13}C-NMR ($CDCl_3$, 100 MHz): δ 172.0, 148.1, 129.3, 125.6, 114.1, 39.9 (bs), 35.6 (bs) ppm. Purified by column chromatography (99:1 DCM:MeOH), R_f= 0.29 with 95:5 DCM:MeOH. mp: 149-150 °C (lit. 151-152 °C). Spectral data were consistent with literature reports.[15]

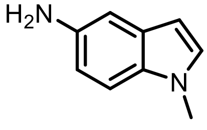

1-methyl-1H-indol-5-amine (2m)

19.2 mg (66%) was isolated as a brown solid following a modified procedure and workup 2 with DCM. A 3:17 mixture of DCE and cyclohexane was used as the solvent. ^{1}H-NMR ($CDCl_3$, 400 MHz): δ 7.13 (d, J = 8.6 Hz, 1H), 6.97 (d, J = 3.0 Hz, 1H), 6.93 (dd, J = 2.2, 0.5 Hz, 1H), 6.70 (dd, J = 8.6, 2.2 Hz, 1H), 6.30 (dd, J = 3.0, 0.8 Hz, 1H), 3.73 (s, 3H), 3.49 (bs, 2H) ppm. ^{13}C-NMR ($CDCl_3$, 100 MHz): δ 139.3, 132.0, 129.4, 129.3, 112.6, 109.8, 105.8, 99.6, 33.0 ppm. Purified by column chromatography (1:1 EtOAc:Hex), R_f = 0.10 with 70:30 Hex:EtOAc. mp: 95-98 °C (lit. 99-100 °C). Spectral data were consistent with literature reports.[15]

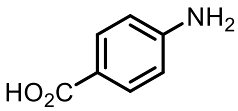

4-aminobenzoic acid (2o)

24.7 mg (90%) was isolated as a white solid from methyl 4-nitrobenzoate following a modified procedure and workup 1. A 3:17 mixture of DCE and cyclohexane was used as the solvent. ^{1}H-NMR (CD_3OD, 500 MHz): δ 7.78 (d, J = 8.7 Hz, 2H), 6.70 (d, J = 8.7 Hz, 2H) ppm. ^{13}C-NMR (CD_3OD, 125 MHz): δ 170.4, 153.2, 132.8, 120.1, 115.1 ppm. Purified by preparatory HPLC using the following method: gradient 5% ACN (H_2O with 0.1% TFA) to 15% ACN (H_2O with 0.1% TFA) over 12 min, 20 mL/min, C18 column; R_T = 4.8 min. mp: 165-166 °C (lit. 184-187 °C). Spectral data were consistent with literature reports.[16] Contains <5% triethylammonium trifluoroate from purification.

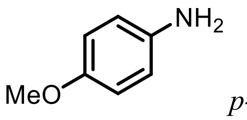

p-anisidine (2p)

4.6 mg (20%) was isolated as a brown solid following the general procedure and workup 2 with EtOAc. ^{1}H-NMR ($CDCl_3$, 400 MHz): δ 6.75 (dt, J = 8.9, 2.4 Hz, 2H), 6.65 (dt, J = 9.0, 2.4 Hz, 2H), 3.75 (s, 3H), 3.42 (bs, 2H) ppm. ^{13}C-NMR ($CDCl_3$, 100 MHz): δ 153.0, 140.1, 116.6, 115.0, 55.9 ppm. Purified by column chromatography (60:39:1 EtOAc:Hex:TEA), R_f = 0.57. mp: 46-48 °C (lit. 56-58 °C). Spectral data were consistent with literature reports.[3]

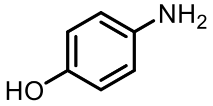

4-aminophenol (2q)

9.2 mg (46%) was isolated as a brown solid following the general procedure and workup 2 with EtOAc. ^{1}H-NMR (CD_3OD, 400 MHz): δ 6.65-6.58 (m, 4H) ppm. ^{13}C-NMR (CD_3OD, 100 MHz): δ 151.3, 140.2, 118.5, 116.7 ppm. Purified by column chromatography (60:39:1 EtOAc:Hex:TEA followed by 95:5 DCM:MeOH), R_f = 0.36. mp: 177-178 °C (lit. 184-186 °C). Spectral data were consistent with literature reports.[17]

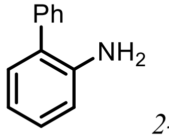

2-aminobiphenyl (2r)

27.0 mg (82%) was isolated as a white solid following the general procedure and workup 1. ^{1}H-NMR ($CDCl_3$, 300 MHz): δ 7.50-7.43 (m, 4H), 7.39-7.34 (m, 1H), 7.21-7.14 (m, 2 H), 6.85 (td, J = 7.4, 1.2 Hz, 1H), 6.79 (ddd, J = 7.9, 1.1, 0.3 Hz, 1H), 3.77 (bs, 2H) ppm. ^{13}C-NMR ($CDCl_3$, 150 MHz): δ 143.6, 139.6, 130.6, 129.2, 128.9, 128.6, 127.7, 127.3, 118.8, 115.7 ppm. Purified by column chromatography (90:9:1 Hex:EtOAc:TEA), R_f = 0.35. mp: 41-42 °C (lit. 47-49 °C). Spectral data were consistent with literature reports.[14]

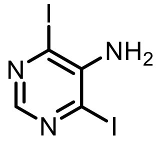

4,6-diiodo-5-aminopyrimidine (2s)

40.8 mg (60%) was isolated as a yellow-brown solid from 4,6-dichloro-5-nitropyrimidine following a modified procedure and workup 2 with EtOAc. A 3:17 mixture of DCE and cyclohexane was used as the solvent, and the reaction was run for 3 h. ^{1}H-NMR ($CDCl_3$, 400 MHz): δ 7.89 (s, 1H), 4.62 (bs, 2H) ppm. ^{13}C-NMR ($CDCl_3$, 100 MHz): δ 148.1, 145.1, 113.4 ppm. Purified by column chromatography (20:80 EtOAc:Hex), R_f = 0.50. mp: 118-121 °C. Spectral data were consistent with literature reports.[18]

1.9. Spectral data

Figure S3: ^{1}H-NMR of 2b in CD_3OD

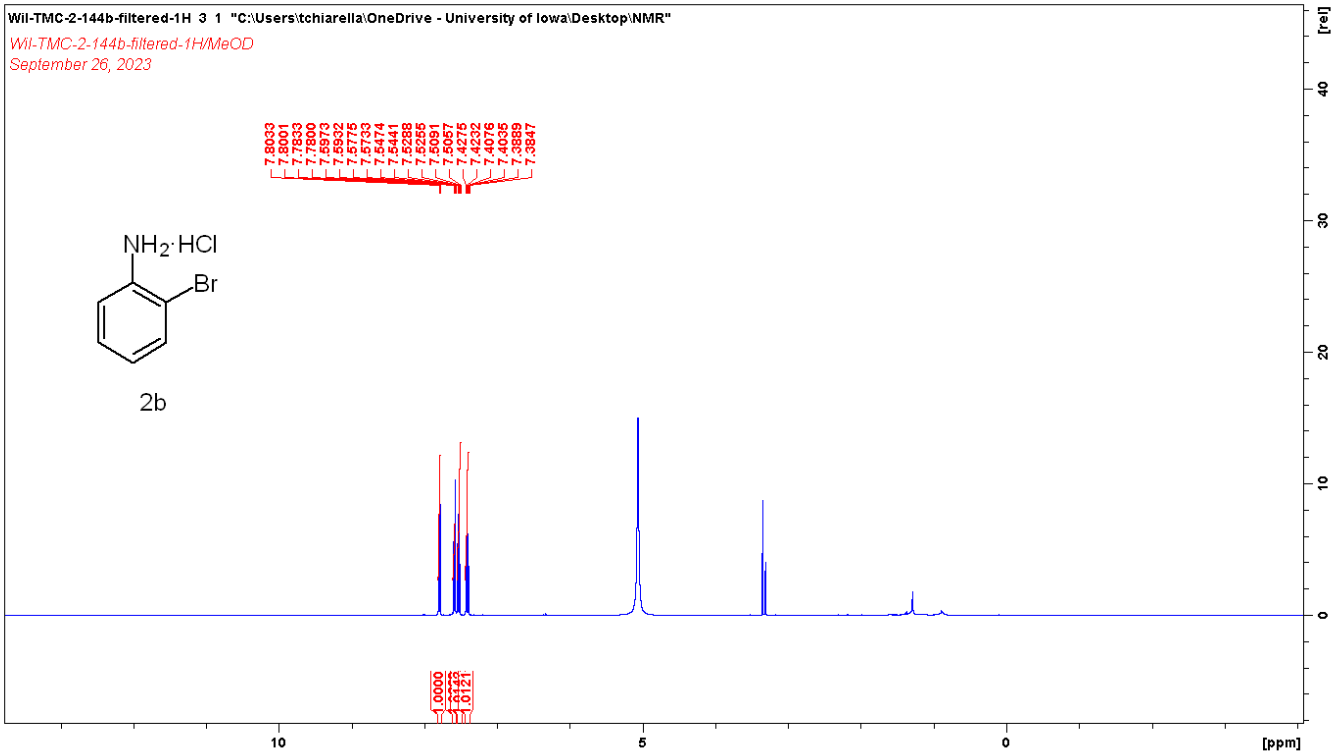

Figure S4: ^{13}C-NMR of 2b in CD_3OD

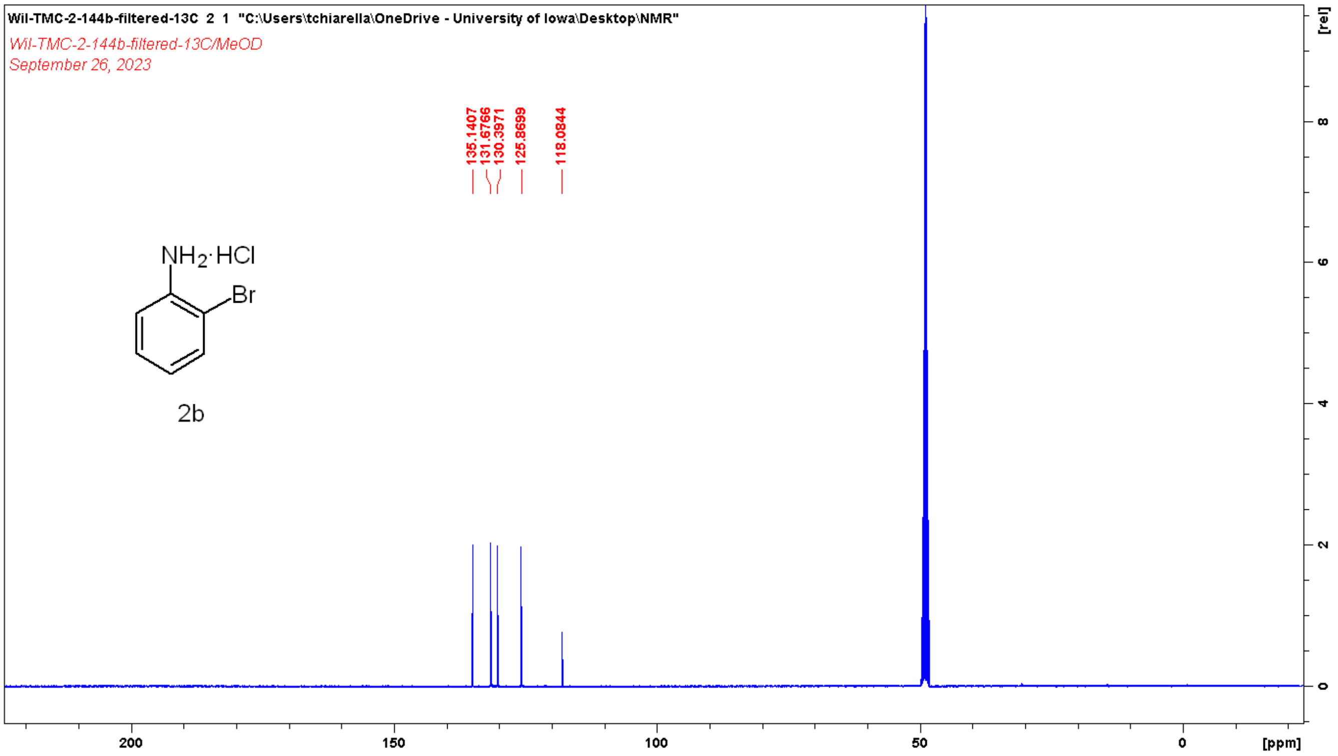

Figure S5: ^{1}H-NMR of 2c in $CDCl_3$

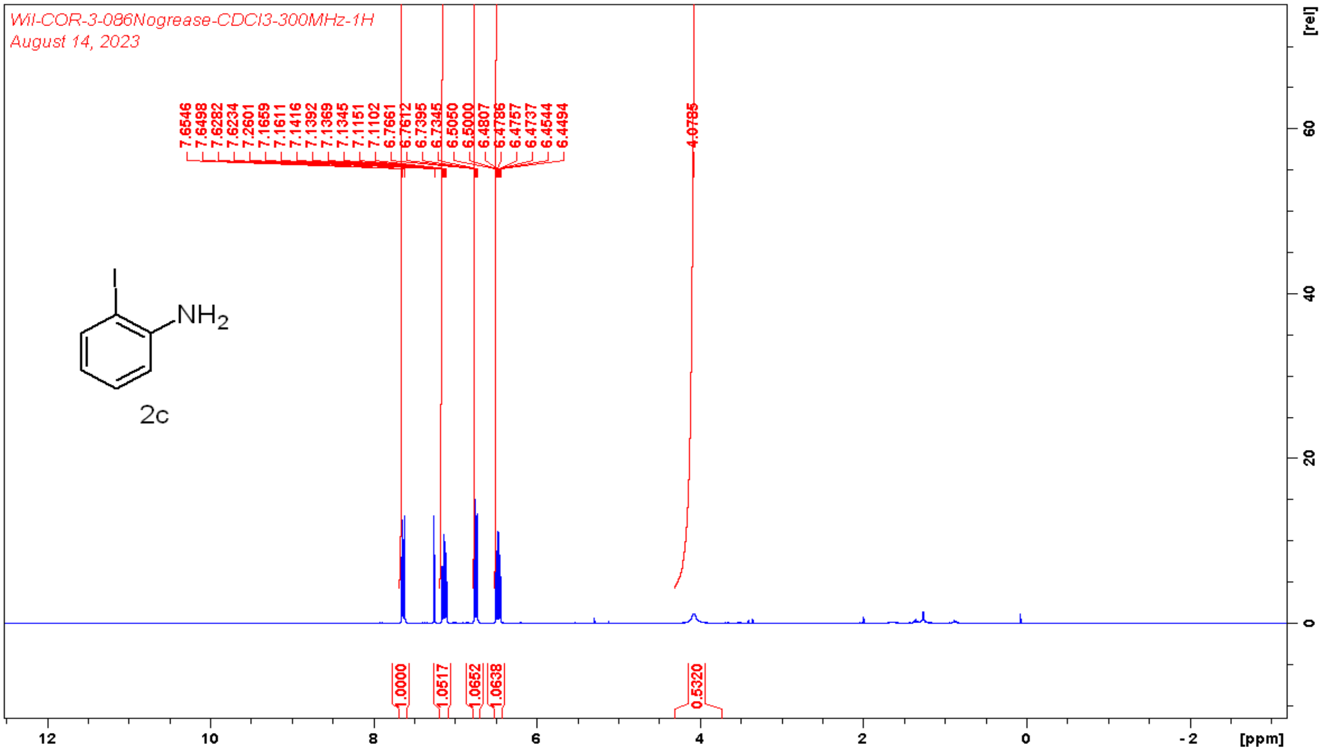

Figure S6: ^{13}C-NMR of 2c in $CDCl_3$

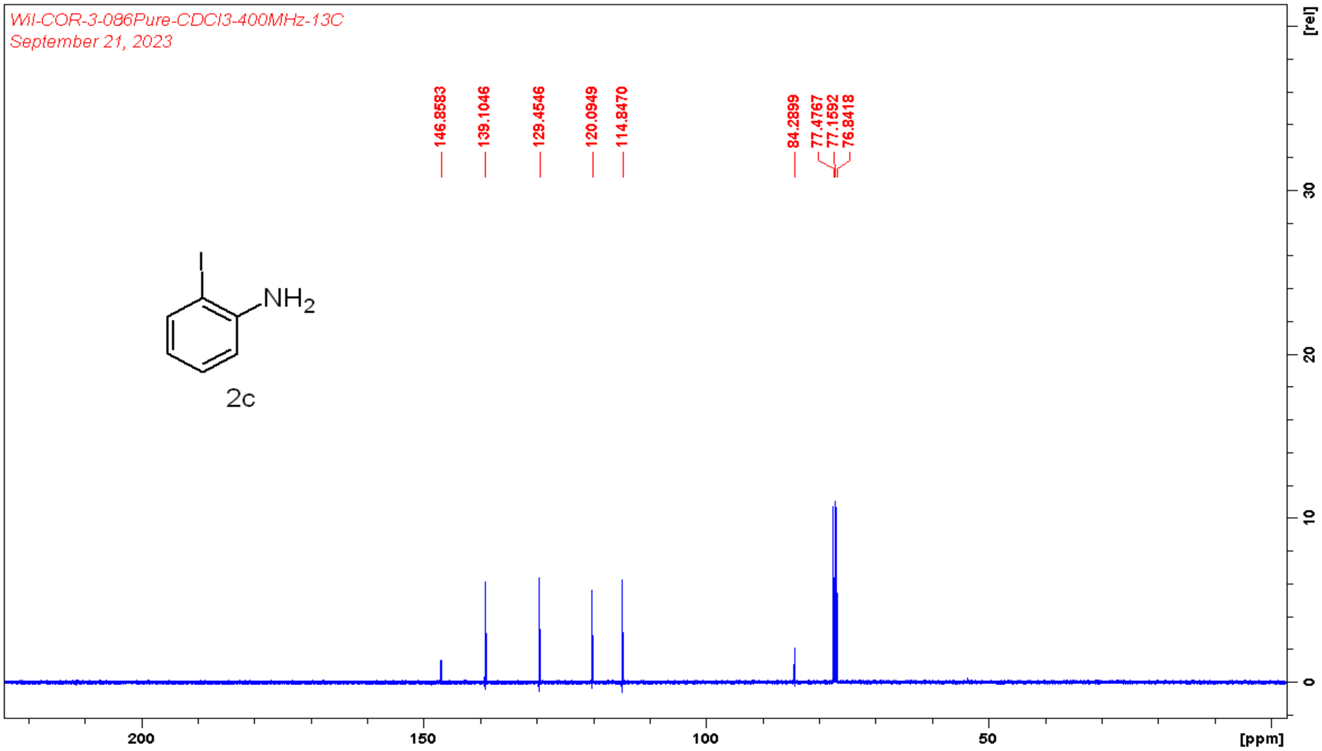

Figure S7: ^{1}H-NMR of 2d in $CDCl_3$

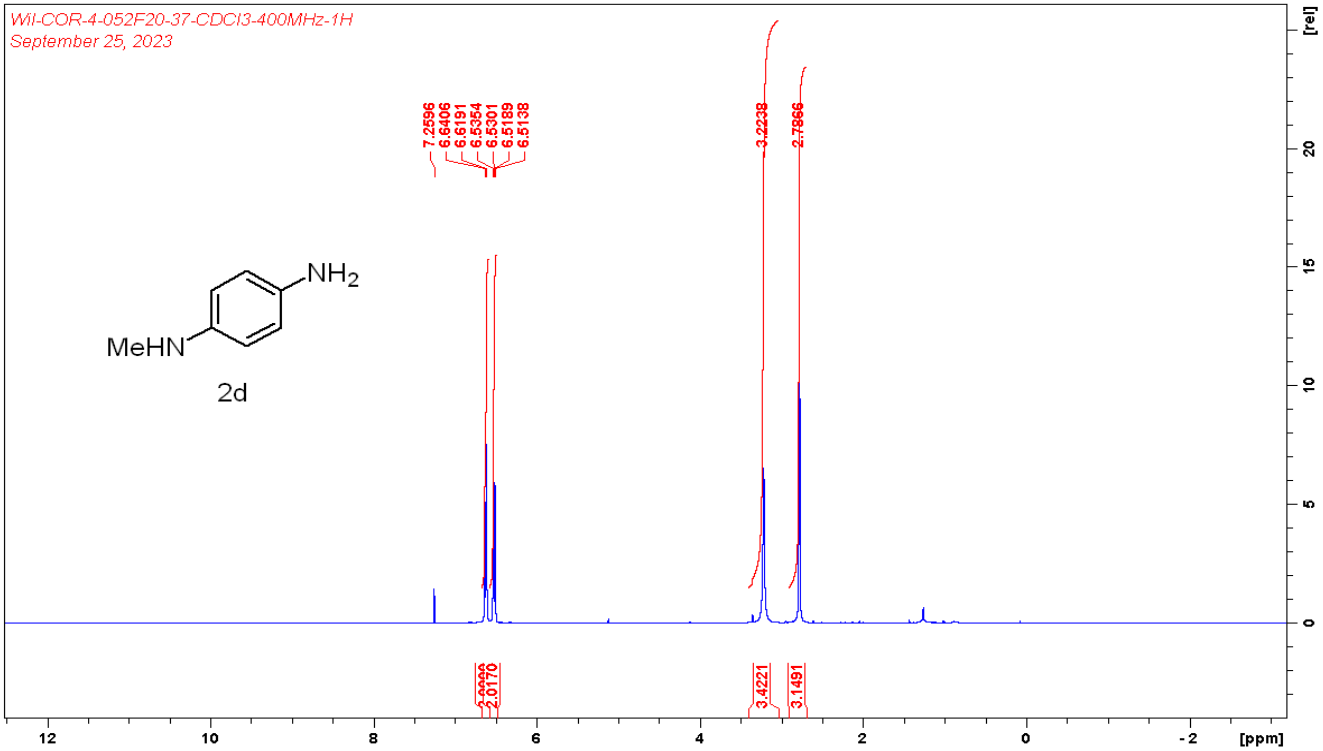

Figure S8: ^{13}C-NMR of 2d in $CDCl_3$

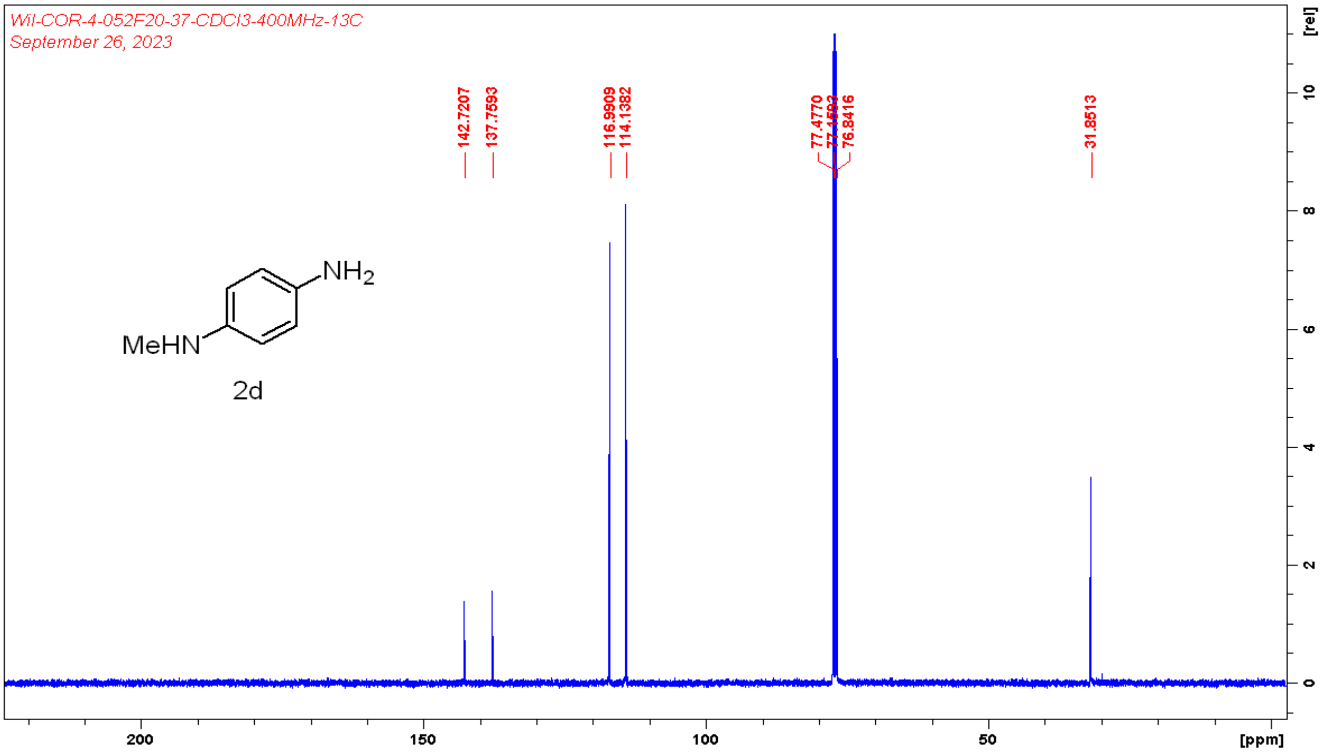

Figure S9: ^{1}H-NMR of 2e in $CDCl_3$

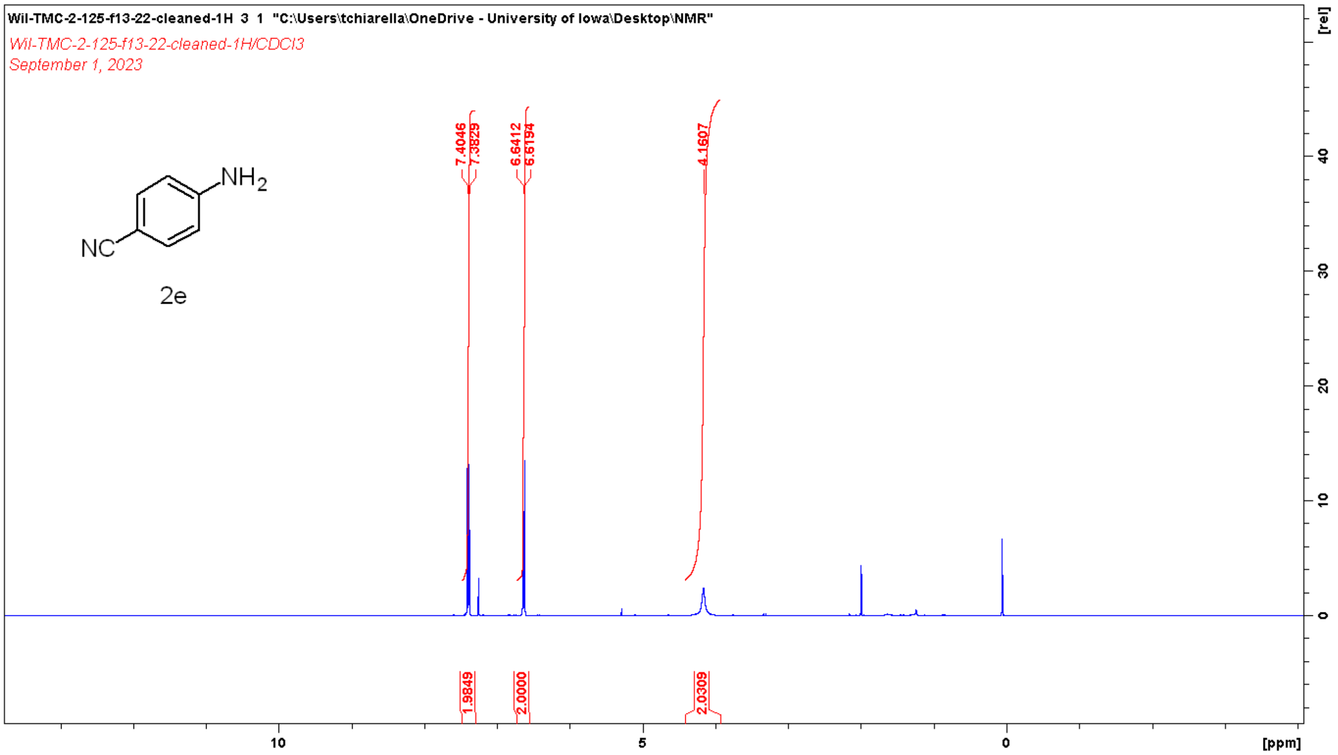

Figure S10: ^{13}C-NMR of 2e in $CDCl_3$

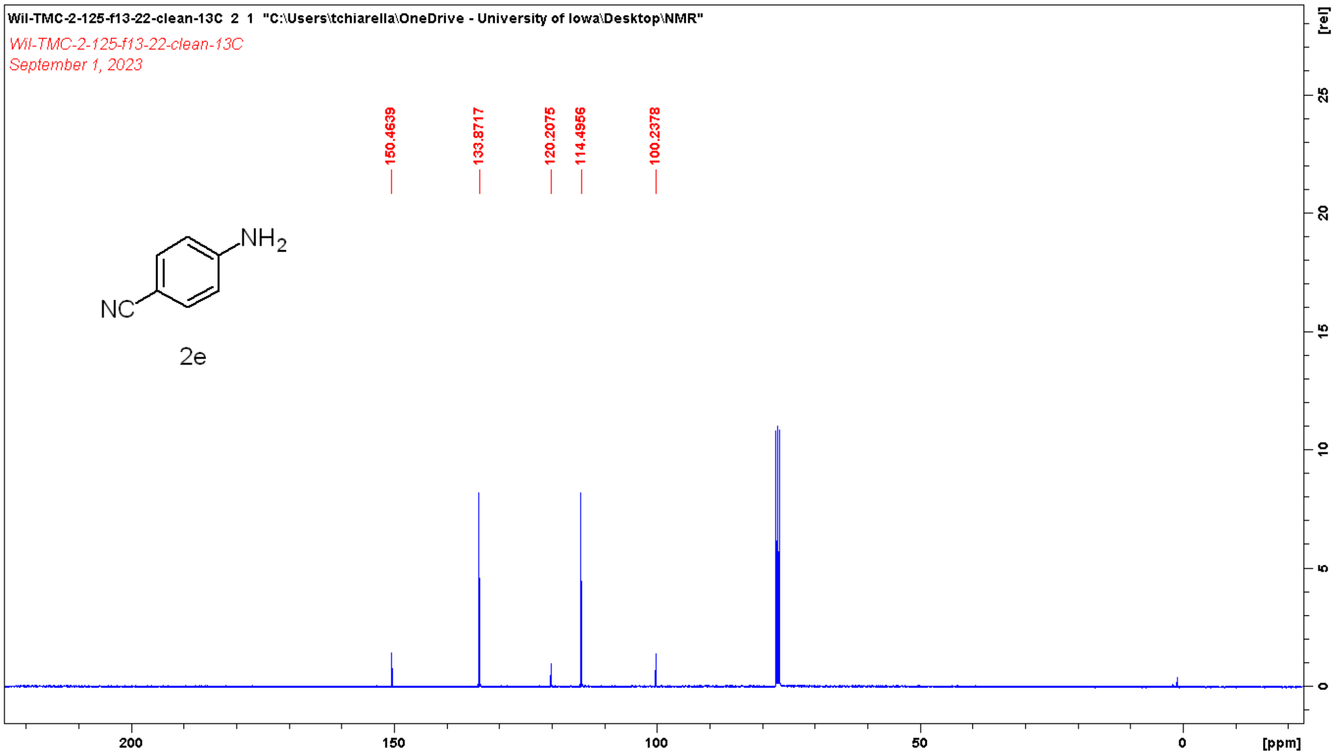

Figure S11: ^{1}H-NMR of 2f in $CDCl_3$

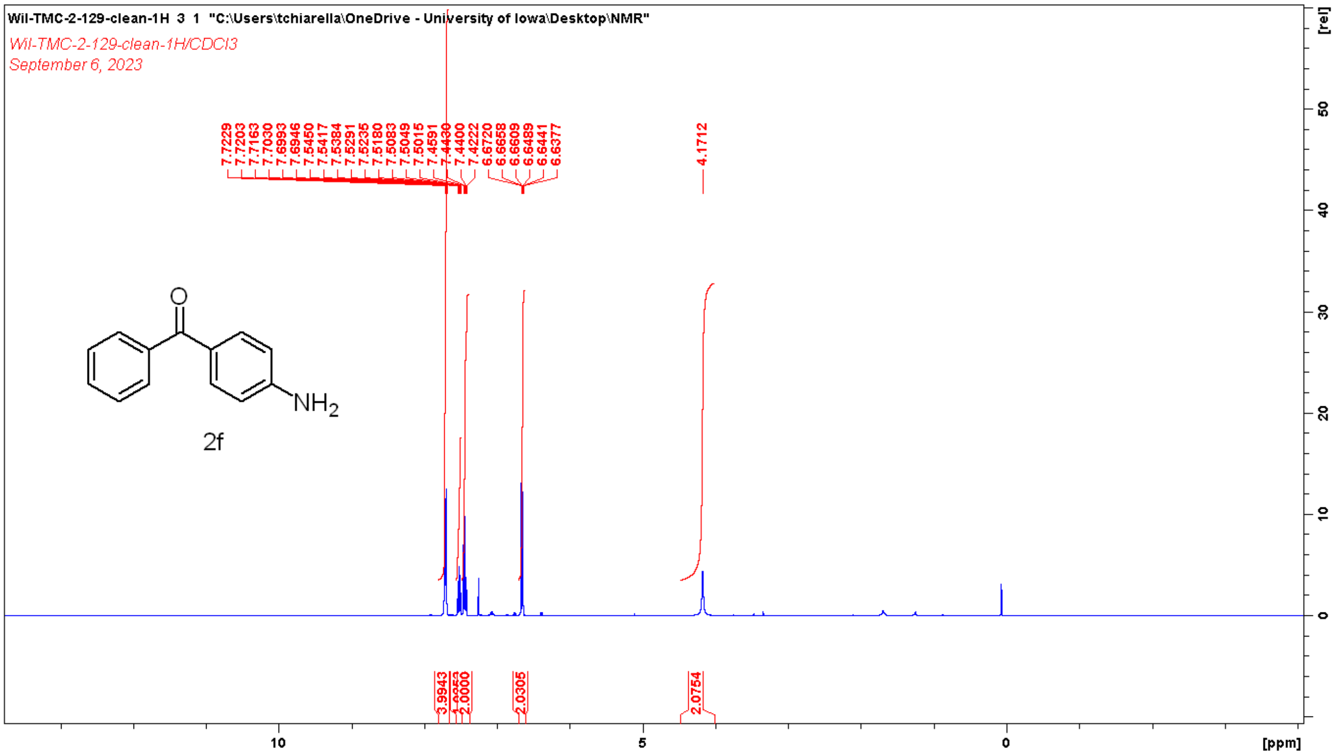

Figure S12: ^{13}C-NMR of 2f in $CDCl_3$

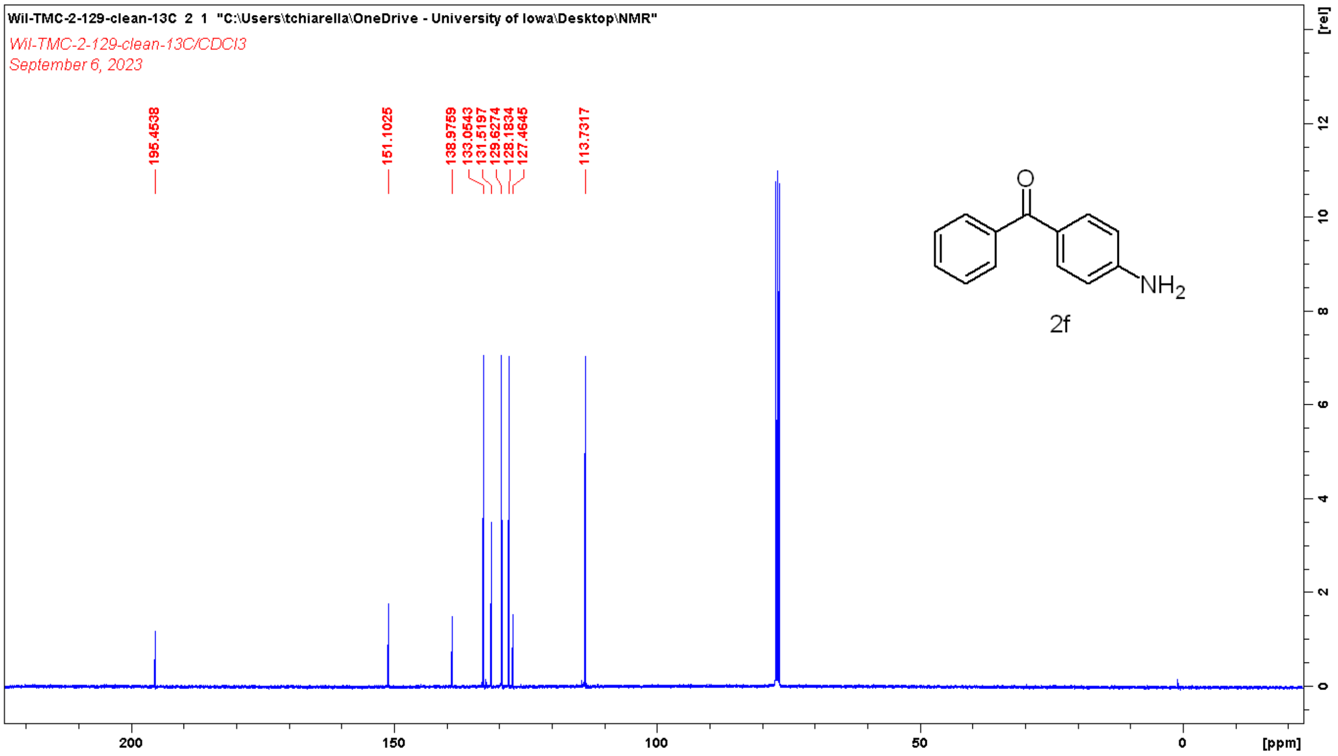

Figure S13: ^{1}H-NMR of 2g in $CDCl_3$

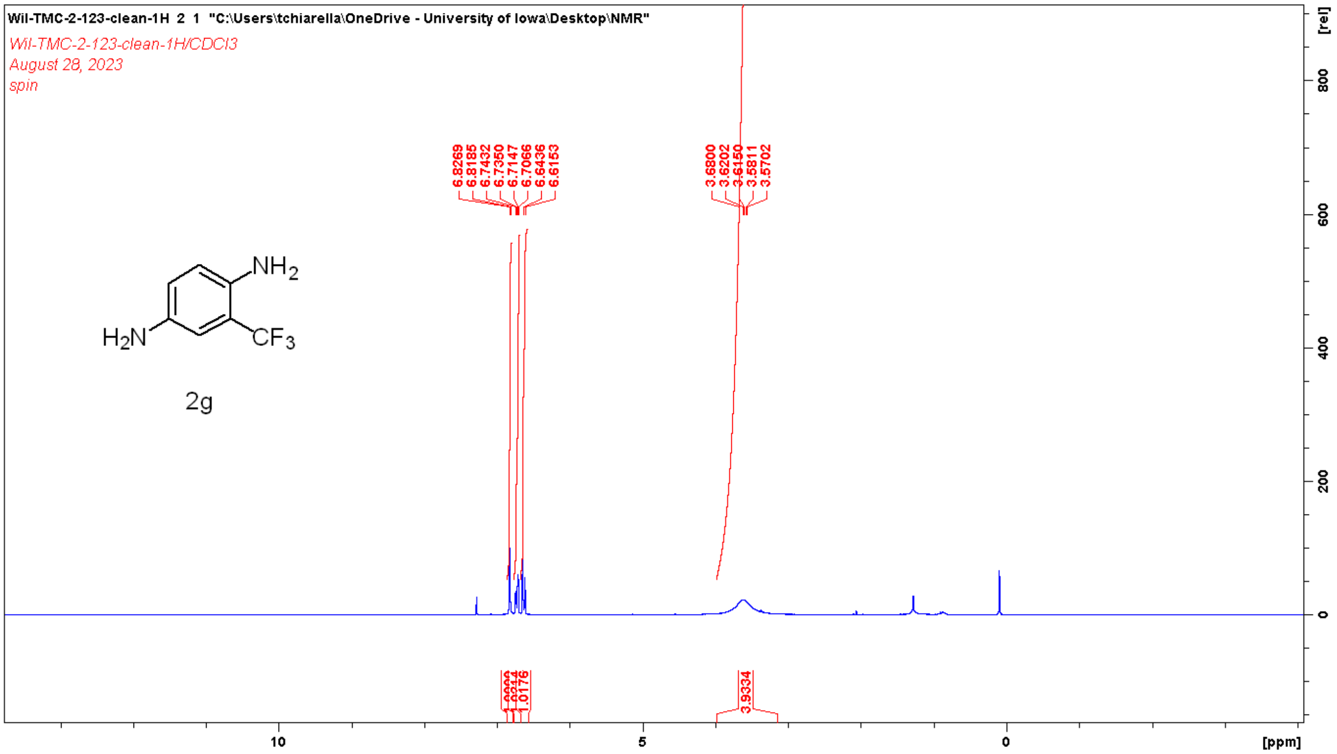

Figure S14: ^{13}C-NMR of 2g in $CDCl_3$

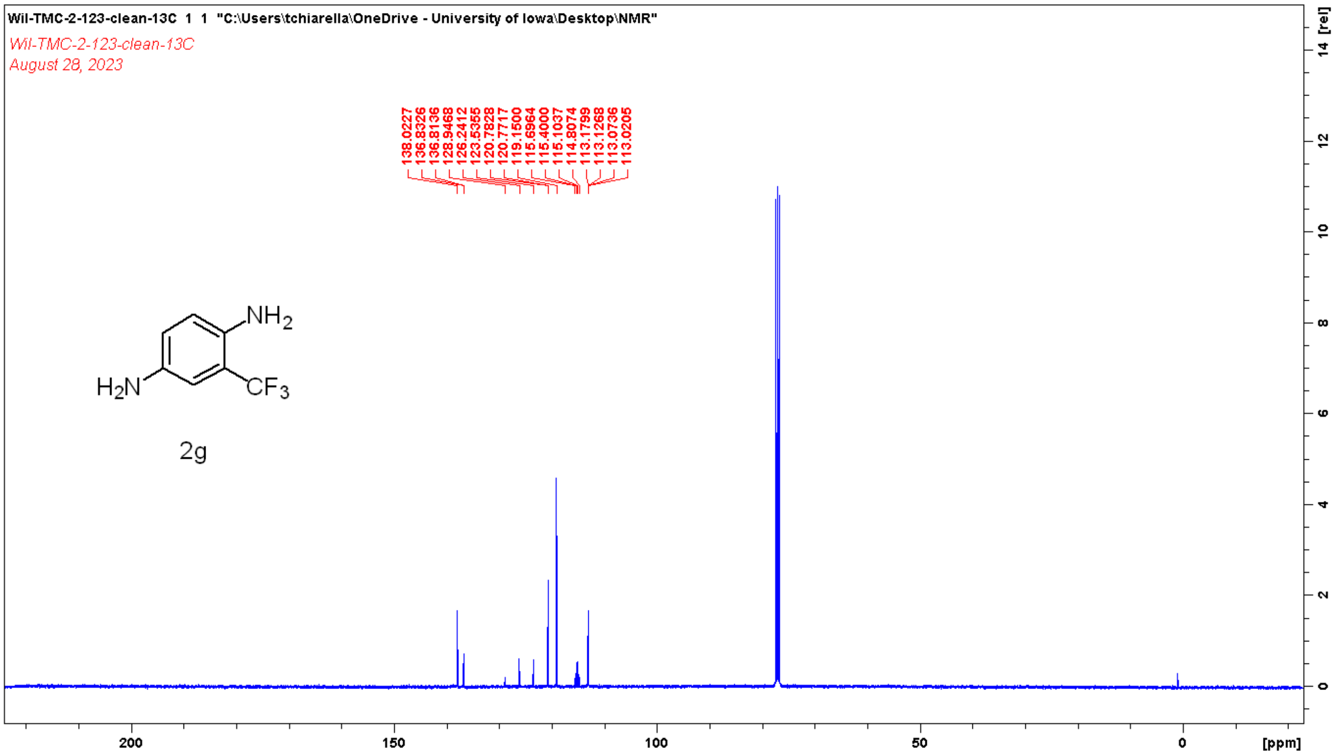

Figure S15: ^{19}F-NMR of 2g in $CDCl_3$, (contains 4-fluorotoluene as an internal standard)

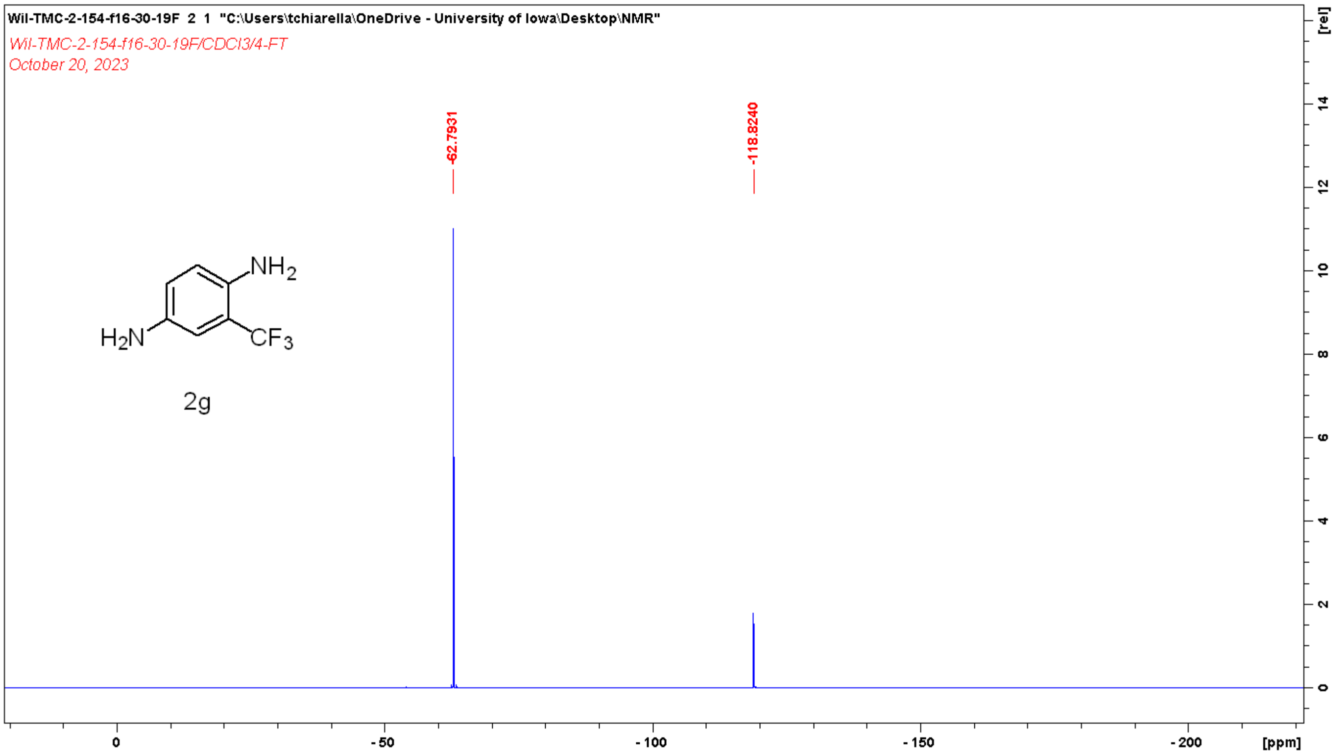

Figure S16: ^{1}H-NMR of 2h in $CDCl_3$

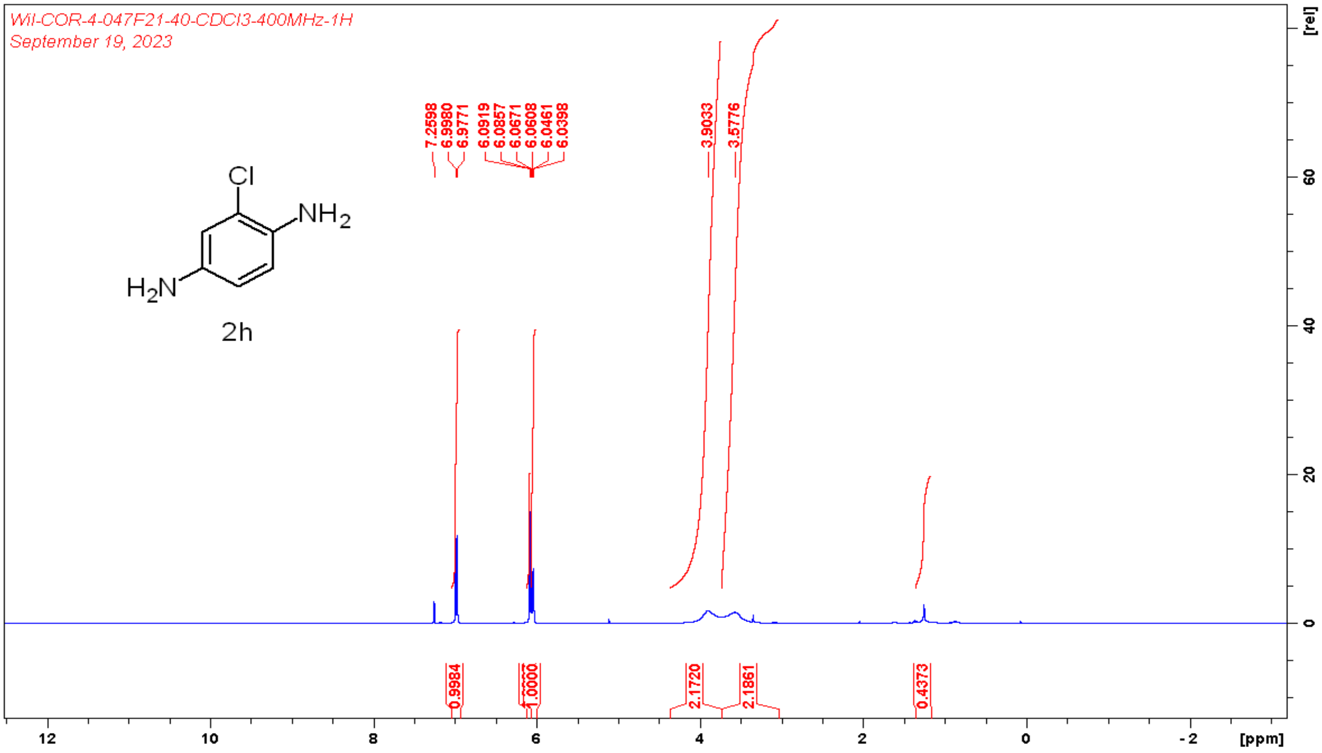

Figure S17: ^{13}C-NMR of 2h in CDCl$_3$

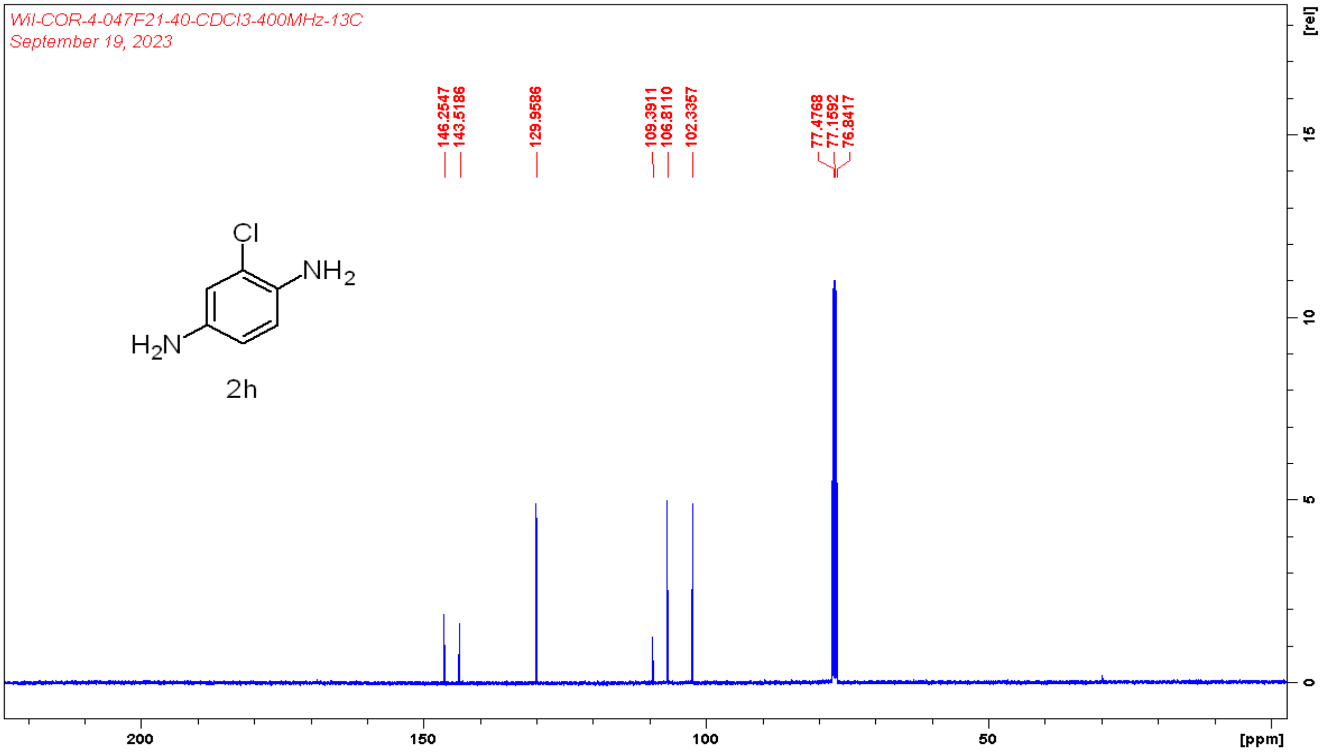

Figure S18: ^{1}H-NMR of 2i in CDCl$_3$

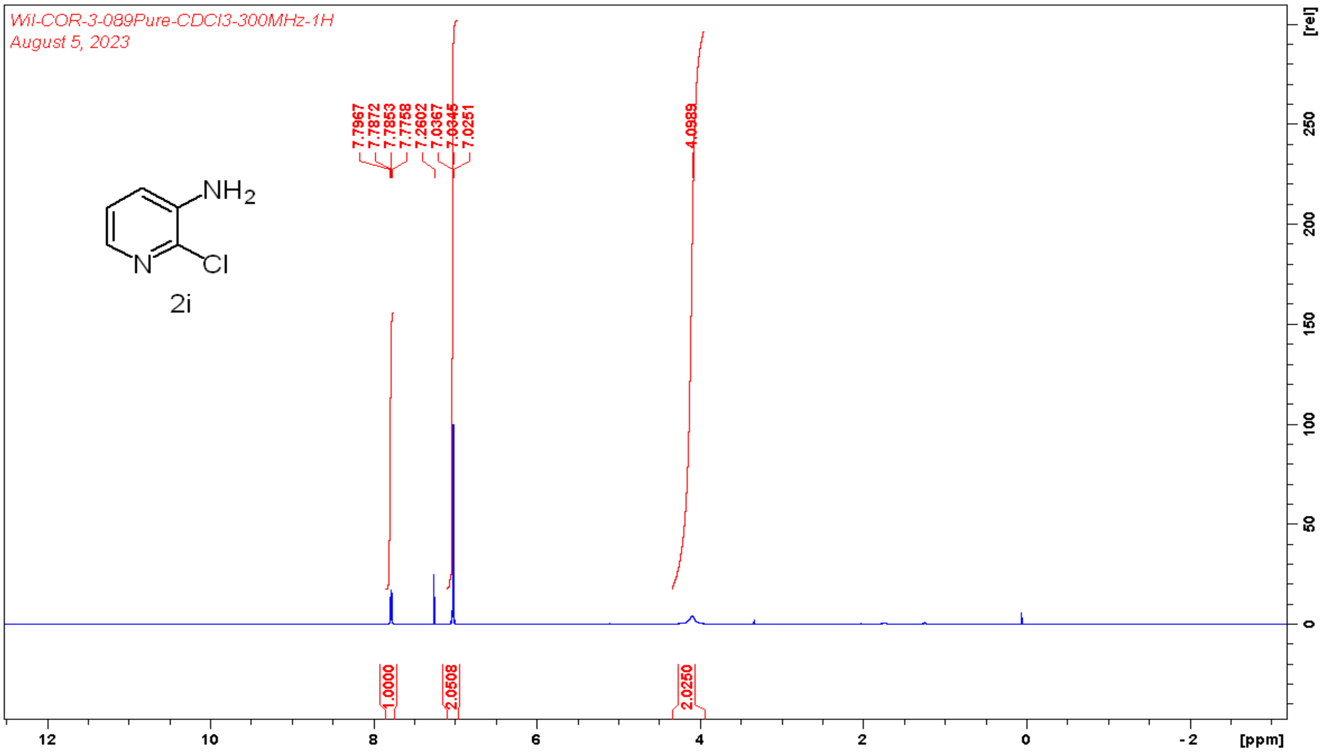

Figure S19: ^{13}C-NMR of 2i in $CDCl_3$

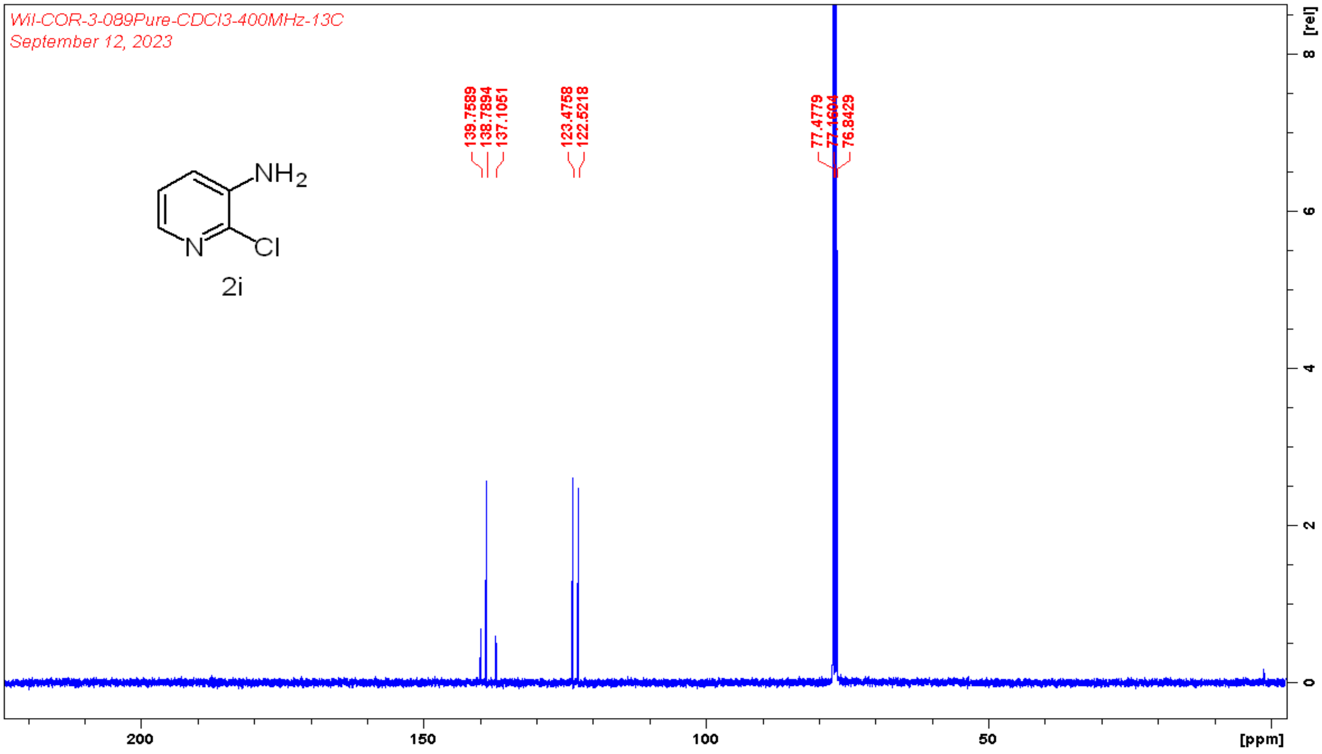

Figure S20: ^{1}H-NMR of 2j in $CDCl_3$ (contains BHT from ether)

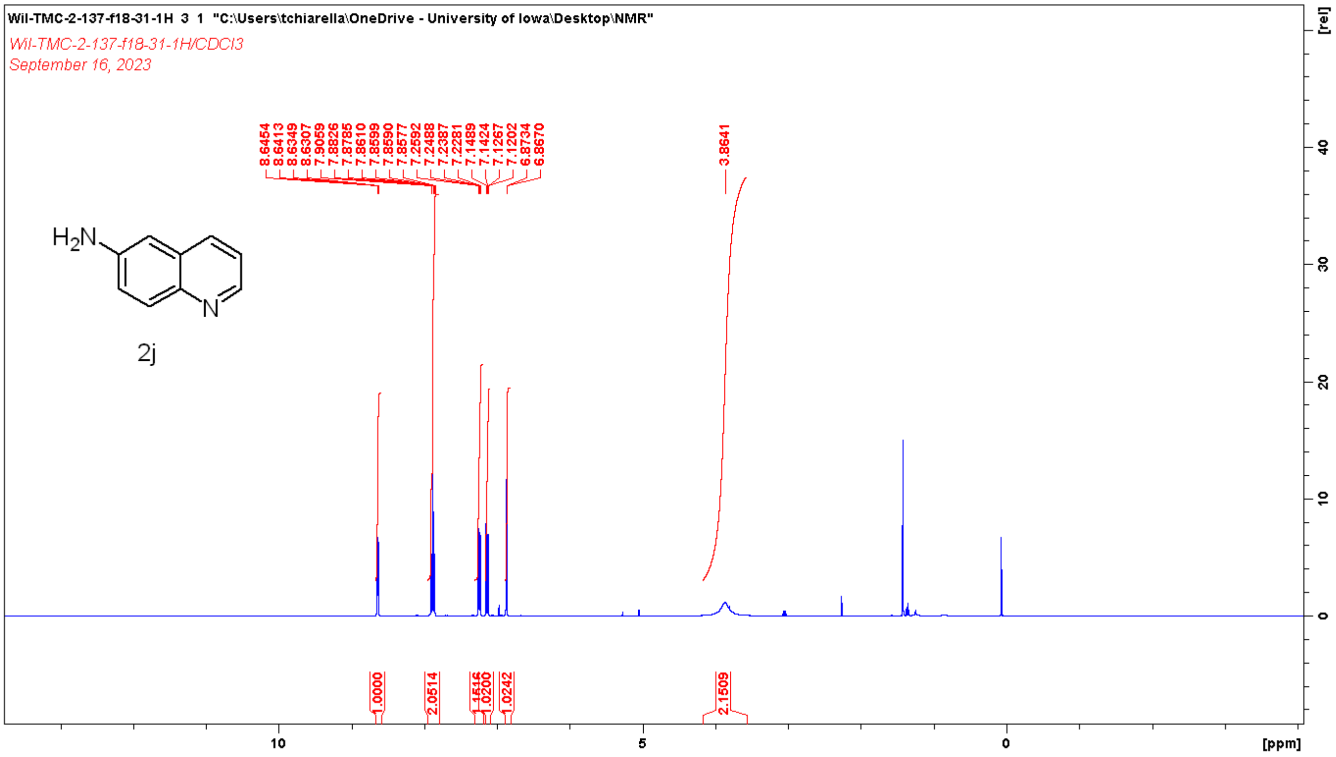

Figure S21: ^{13}C-NMR of 2j in $CDCl_3$ (contains BHT from ether)

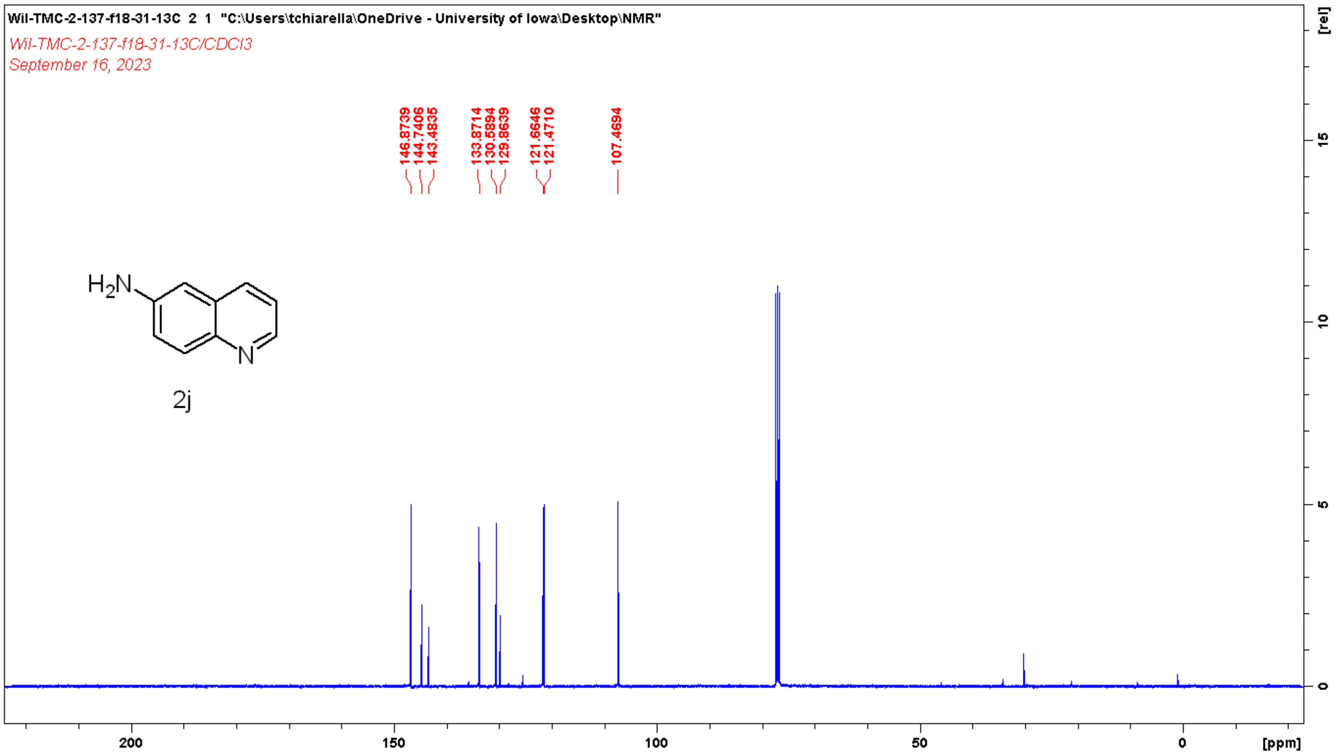

Figure S22: ^{1}H-NMR of 2l in $CDCl_3$

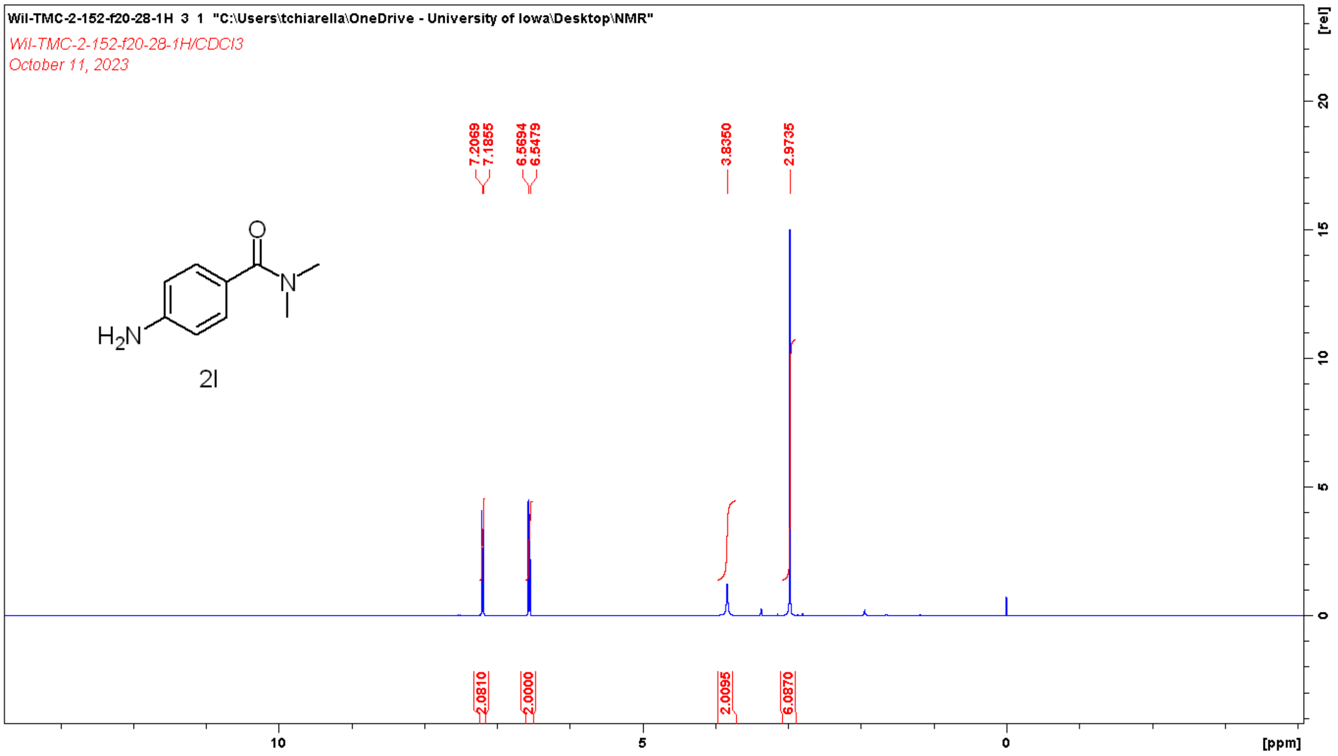

Figure S23: ^{13}C-NMR of 2l in $CDCl_3$

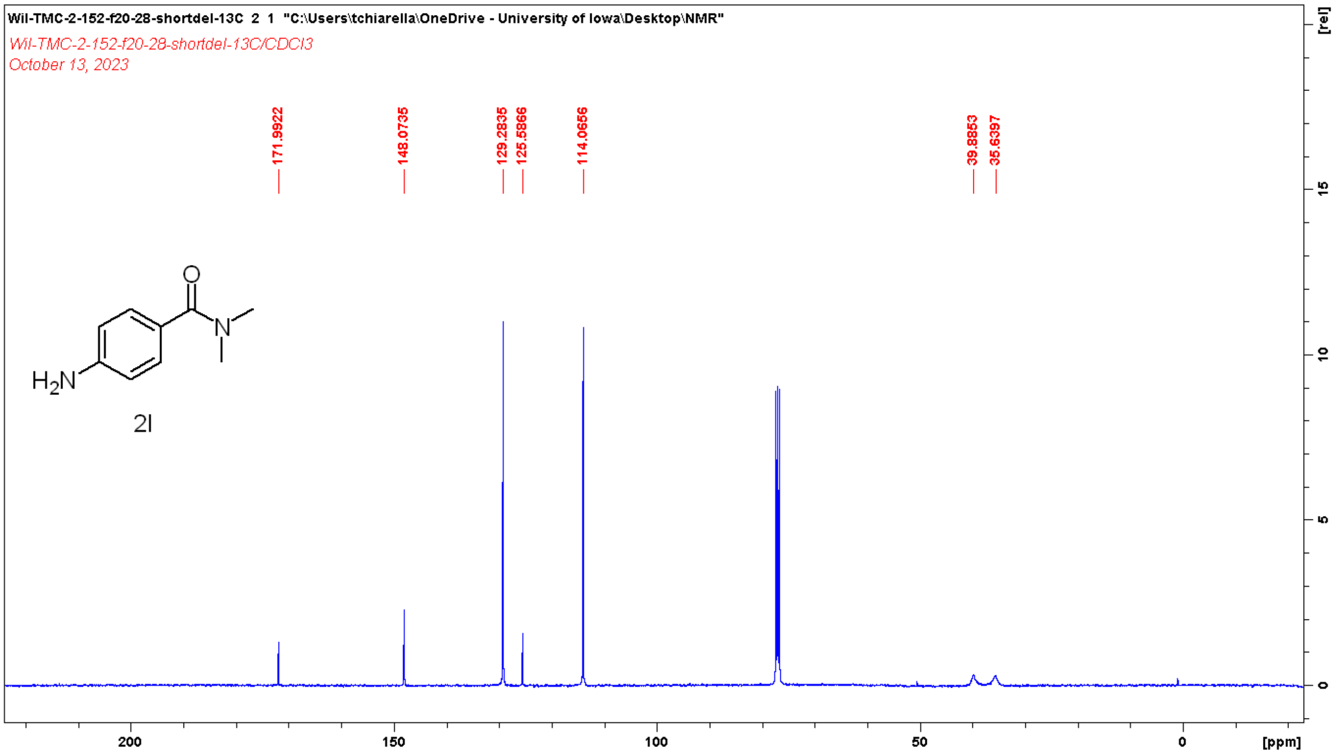

Figure S24: ^{1}H-NMR of 2m in $CDCl_3$

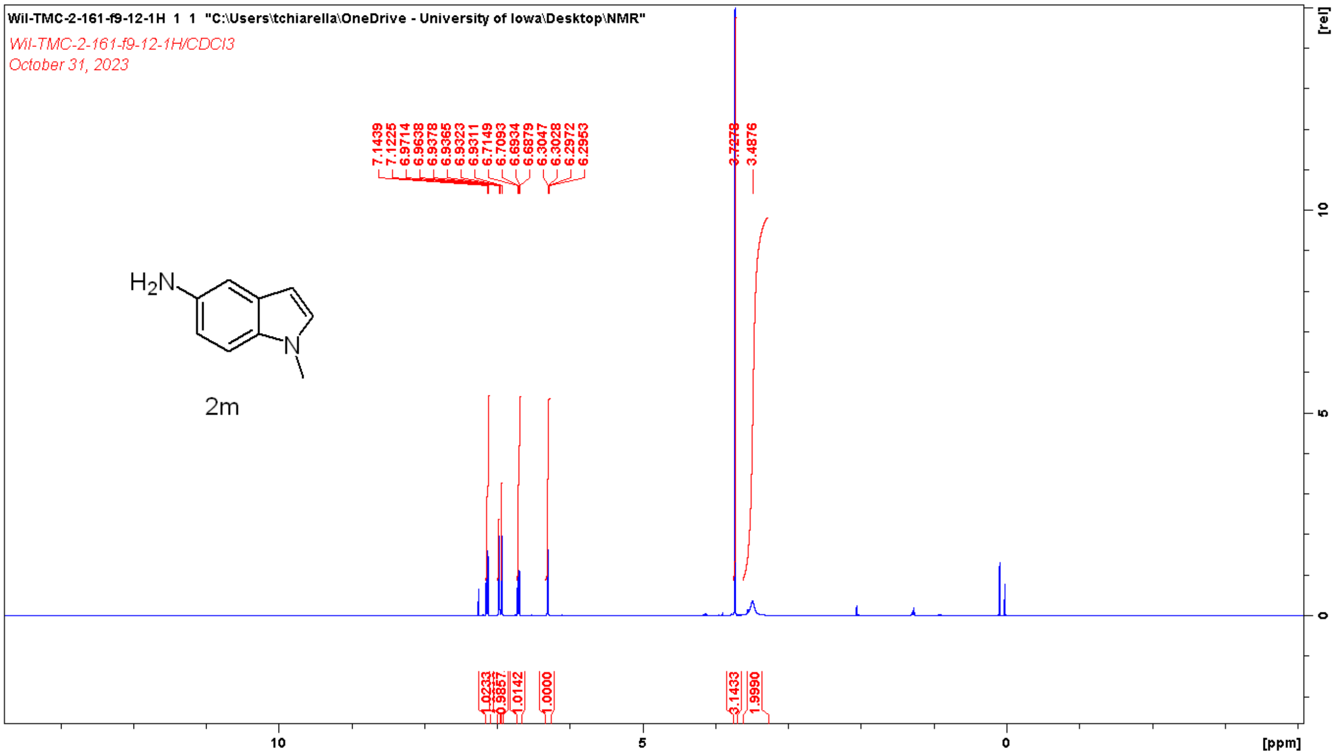

Figure S25: ^{13}C-NMR of 2m in $CDCl_3$

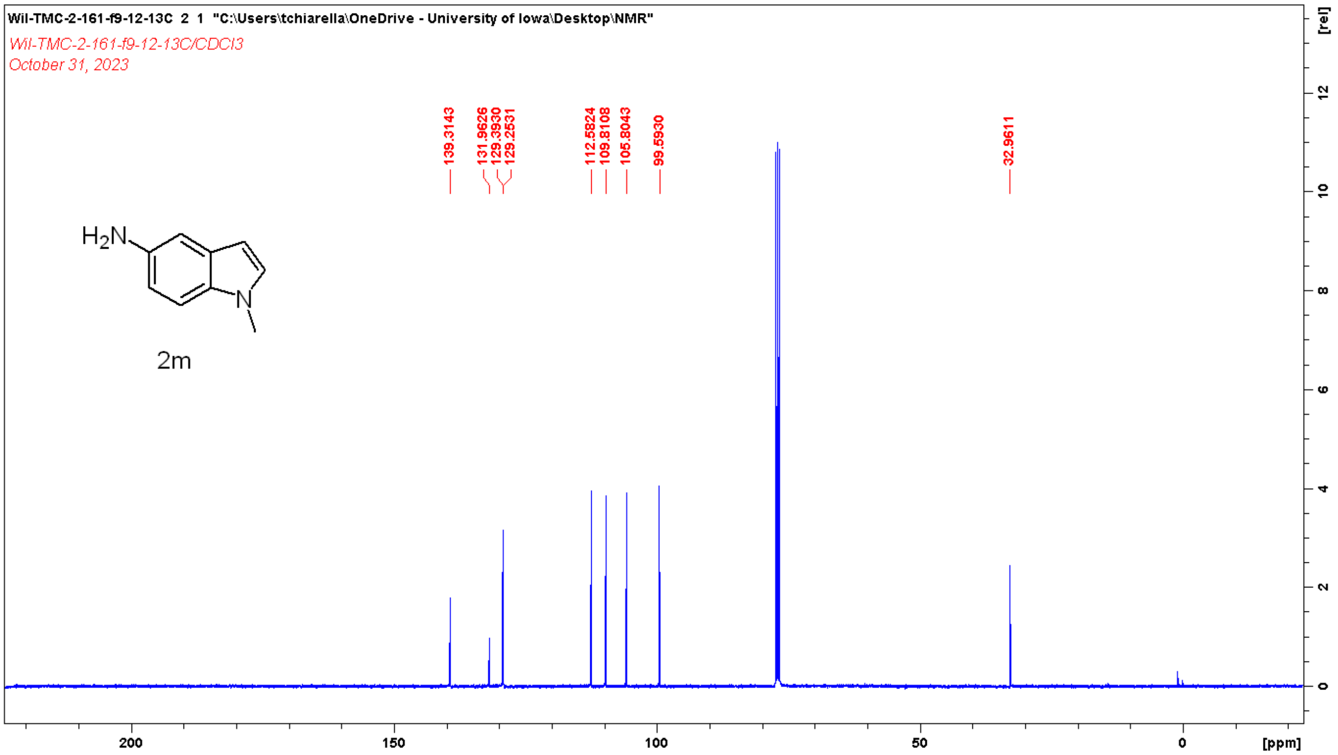

Figure S26: ^{1}H-NMR of 2o in CD_3OD (contains <5% triethylammonium trifluoroate from purification)

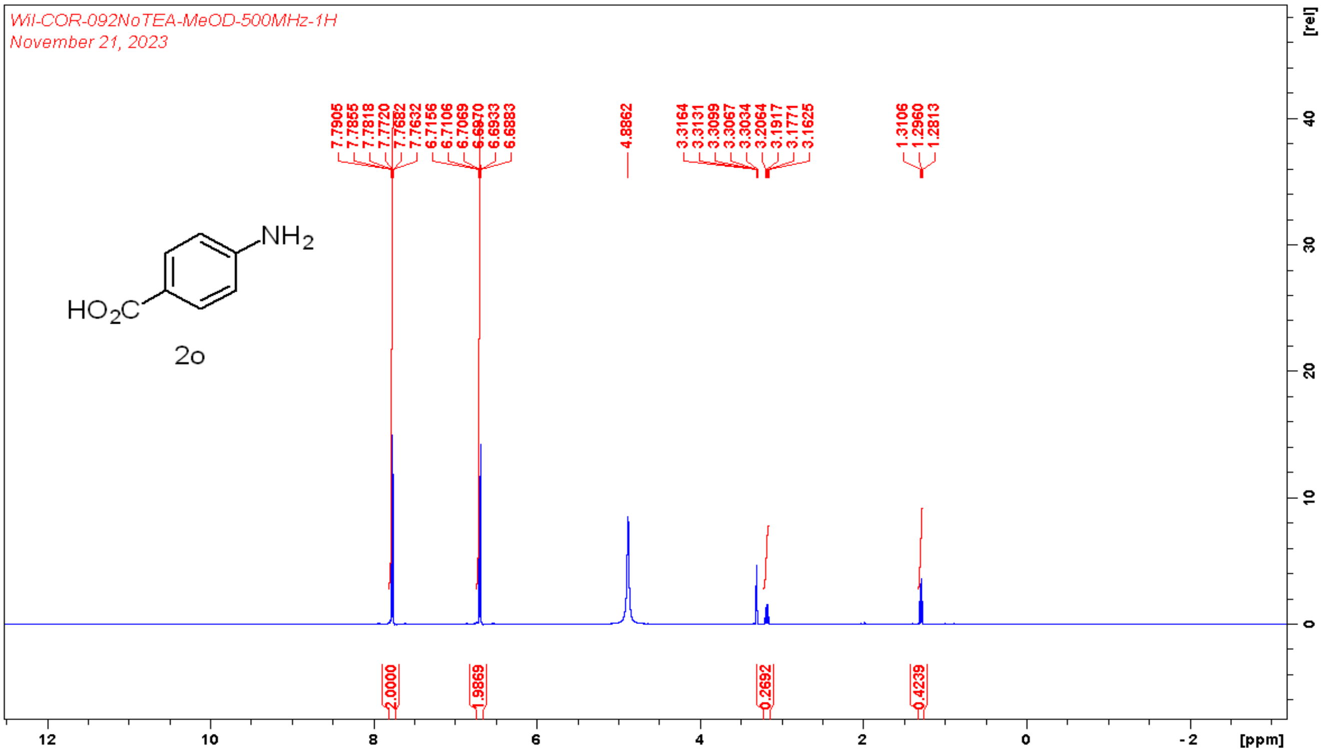

Figure S27: ^{13}C-NMR of 2o in CD_3OD (contains <5% triethylammonium trifluoroate from purification)

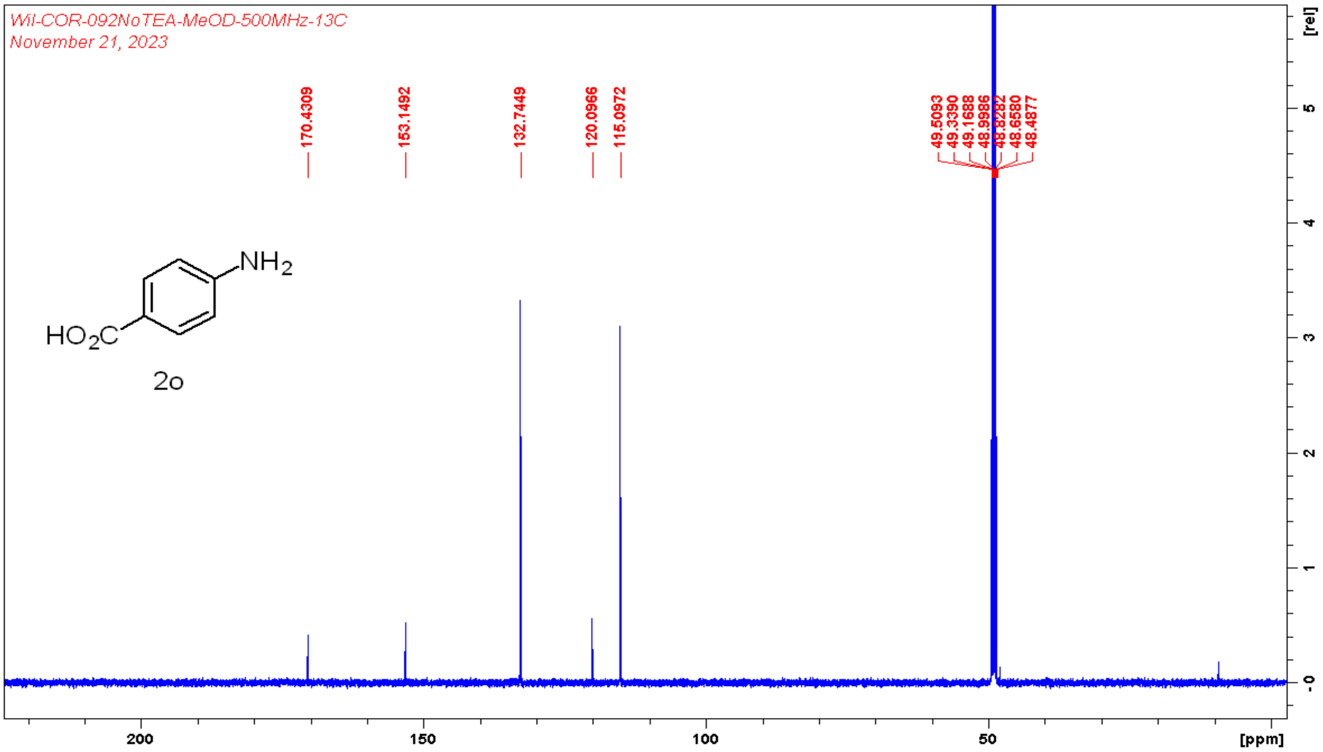

Figure S28: ^{1}H-NMR of 2p in $CDCl_3$

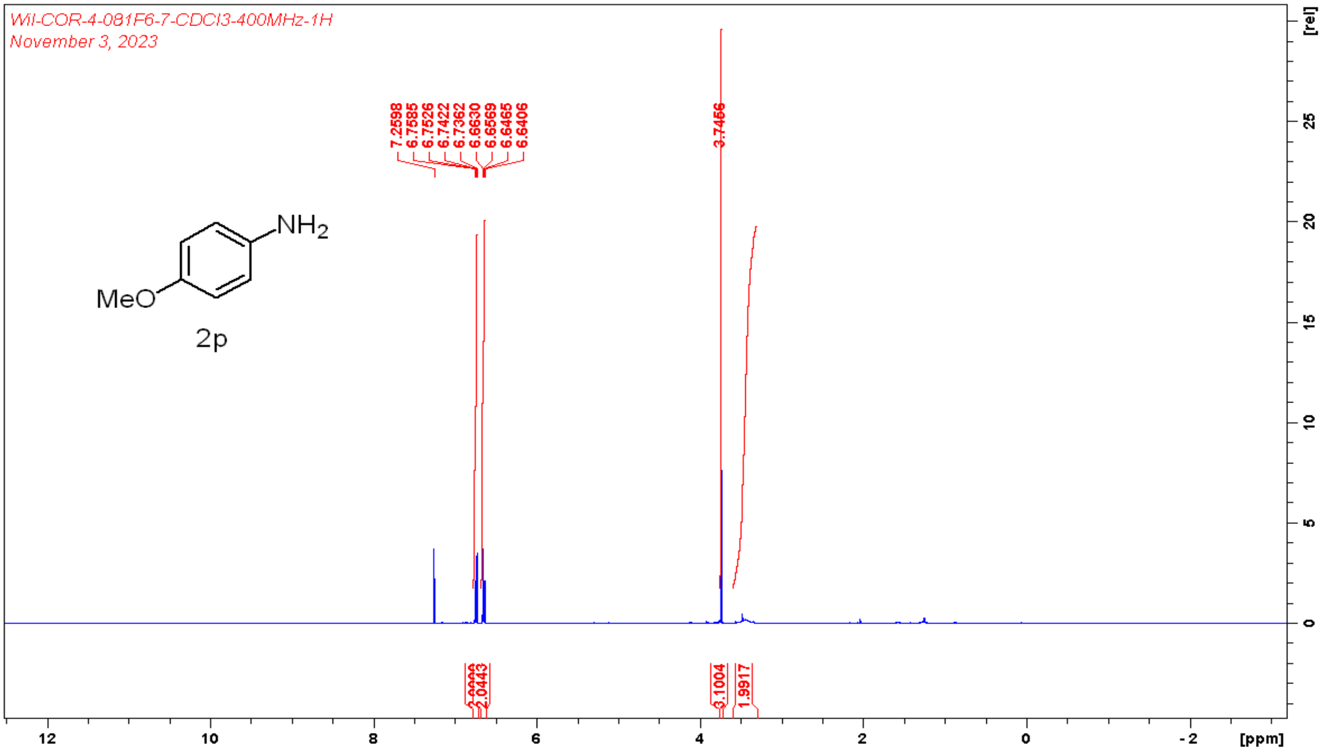

Figure S29: ^{13}C-NMR of 2p in $CDCl_3$

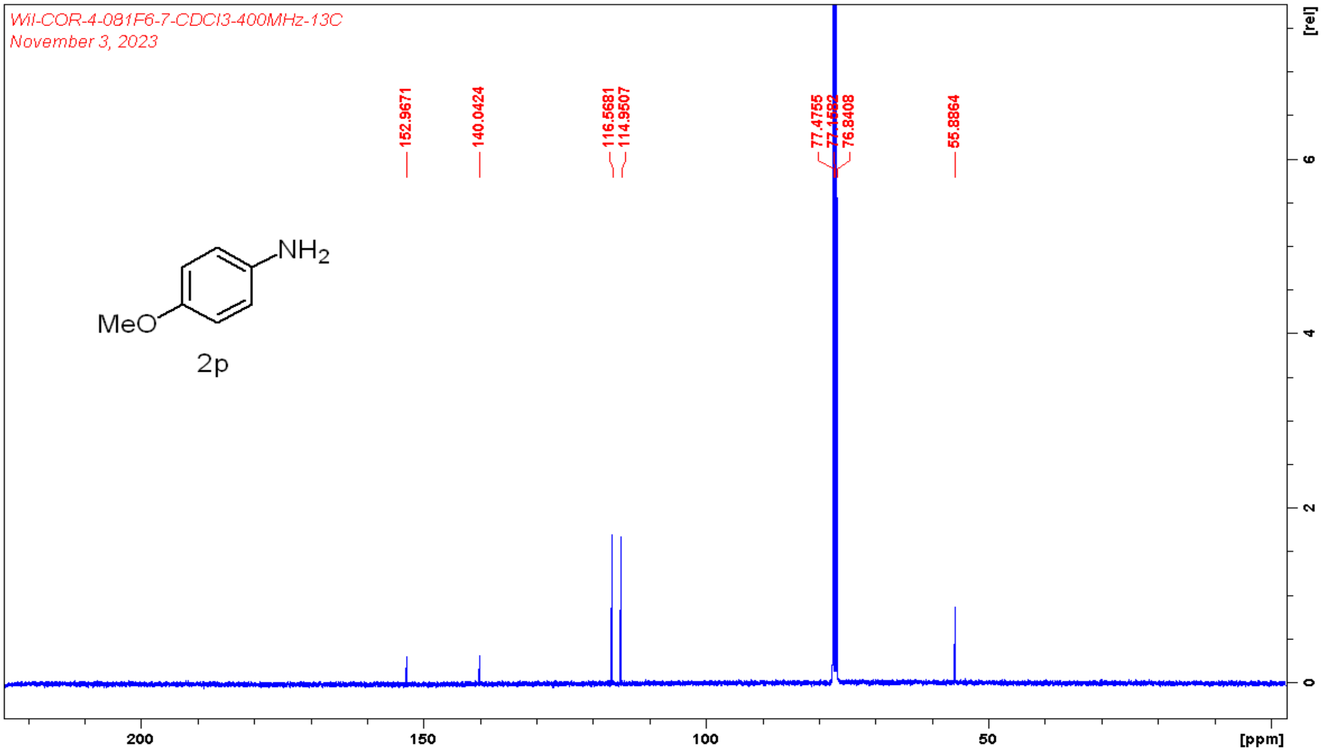

Figure S30: ^{1}H-NMR of 2q in CD_3OD

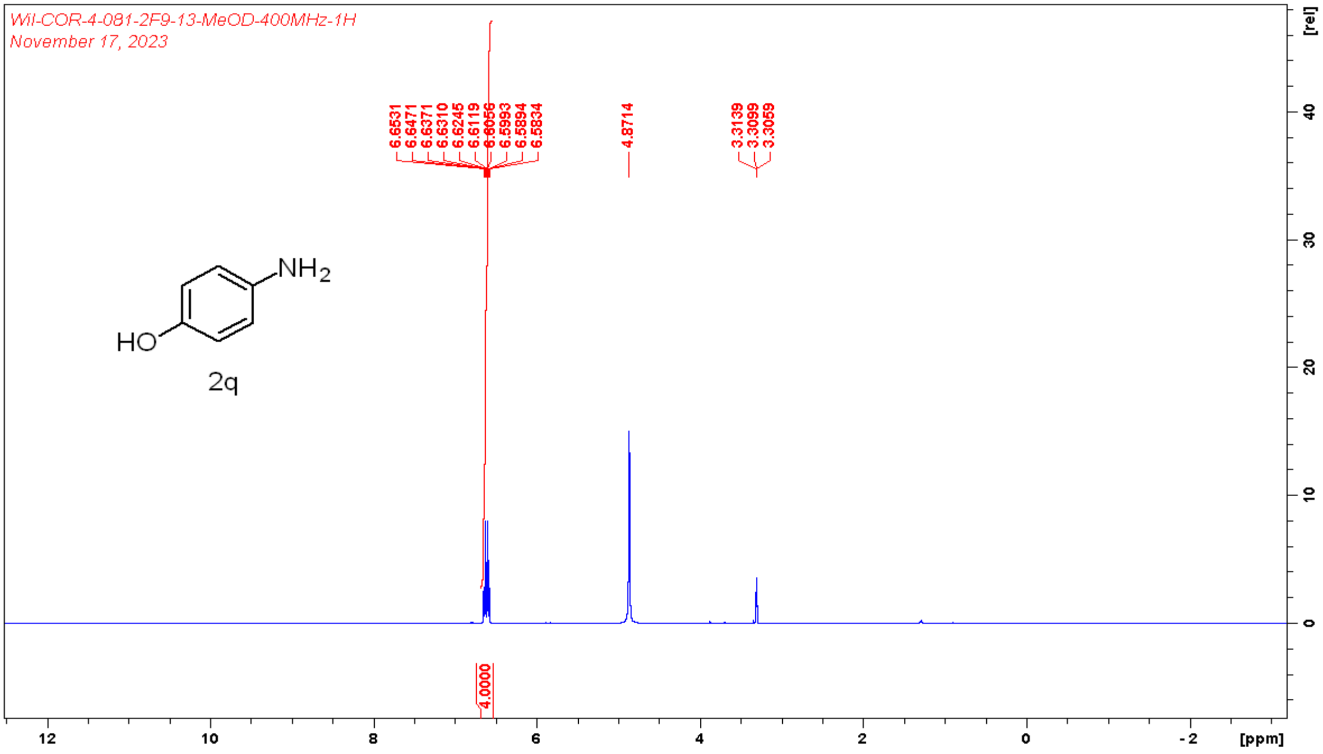

Figure S31: ^{13}C-NMR of 2q in CD_3OD

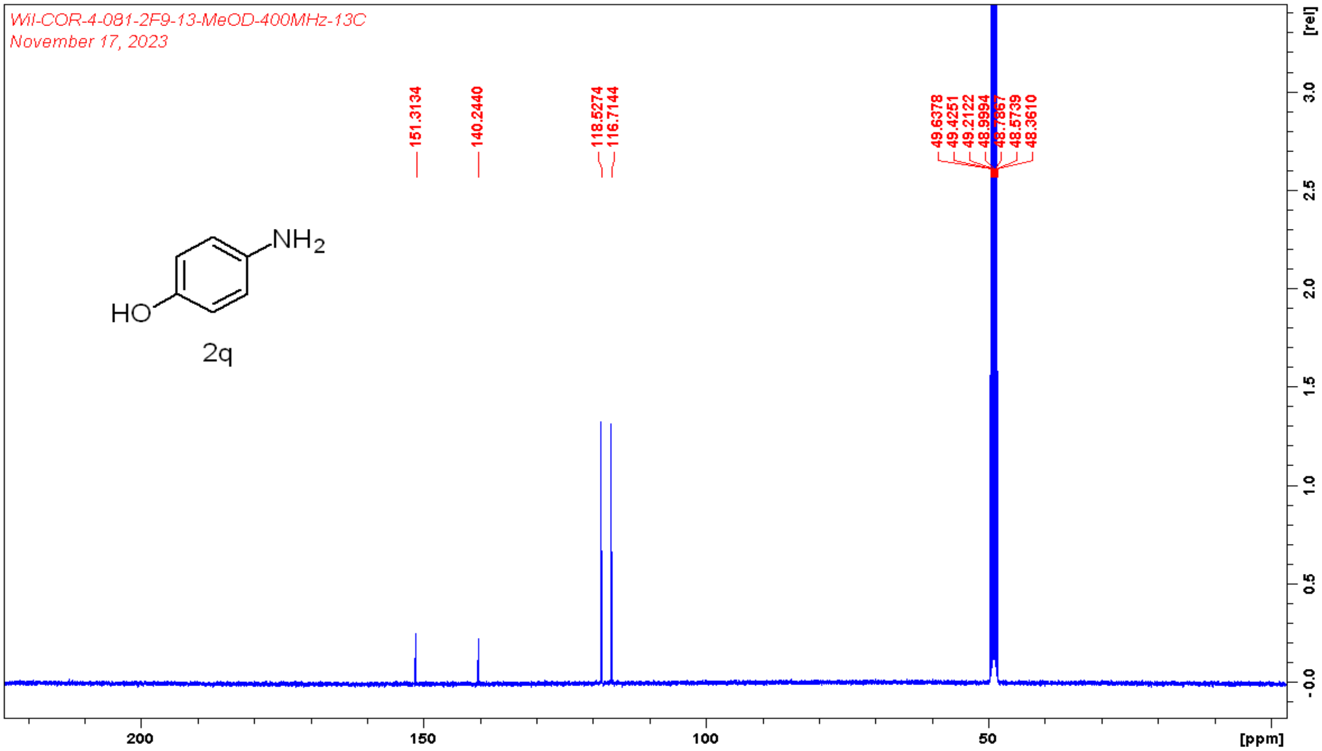

Figure S32: ^{1}H-NMR of 2r in $CDCl_3$

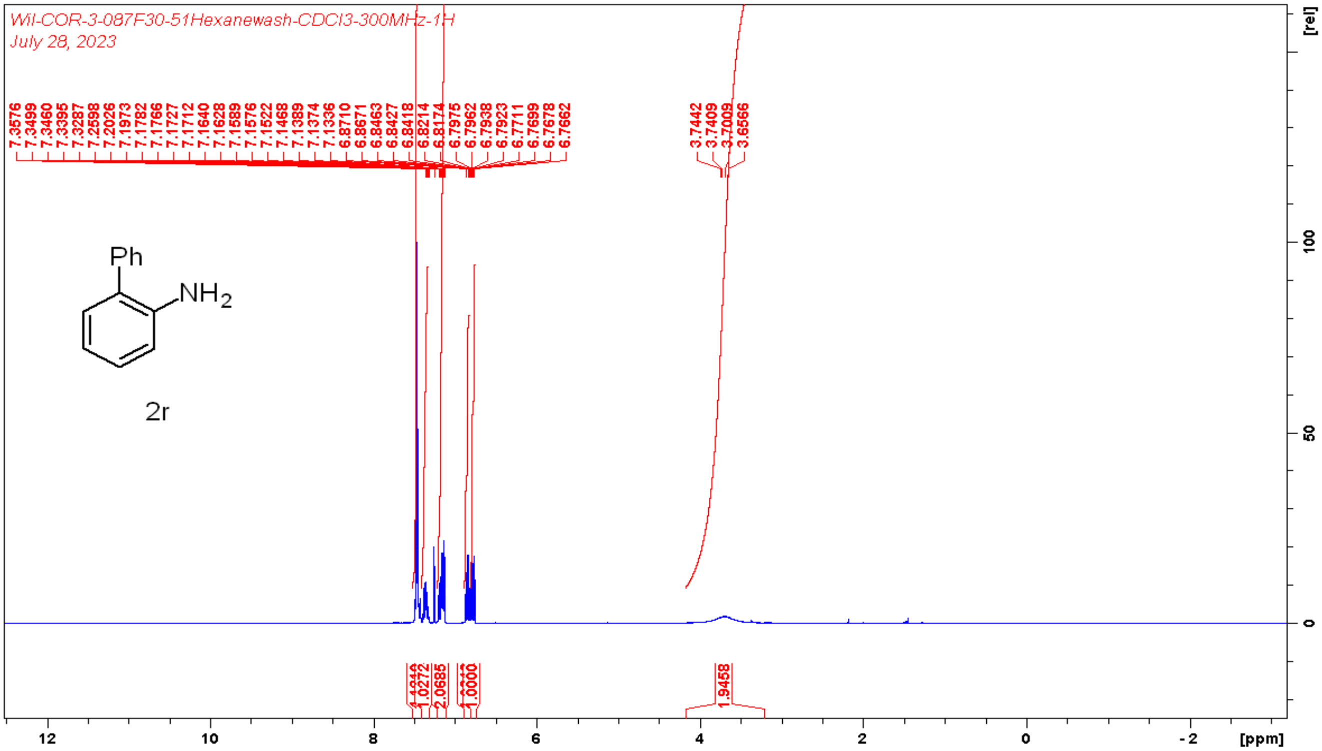

Figure S33: ^{13}C-NMR of 2r in $CDCl_3$

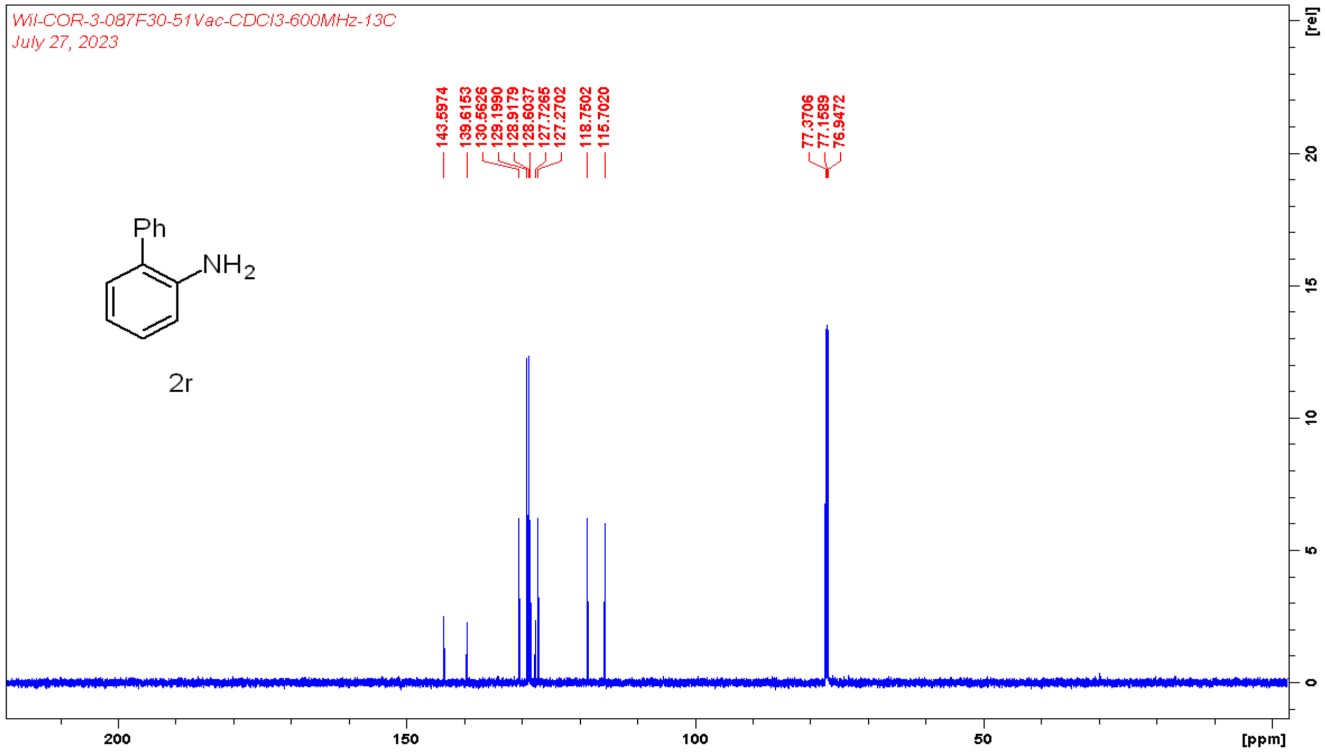

Figure S34: ^{1}H-NMR of 2s in $CDCl_3$

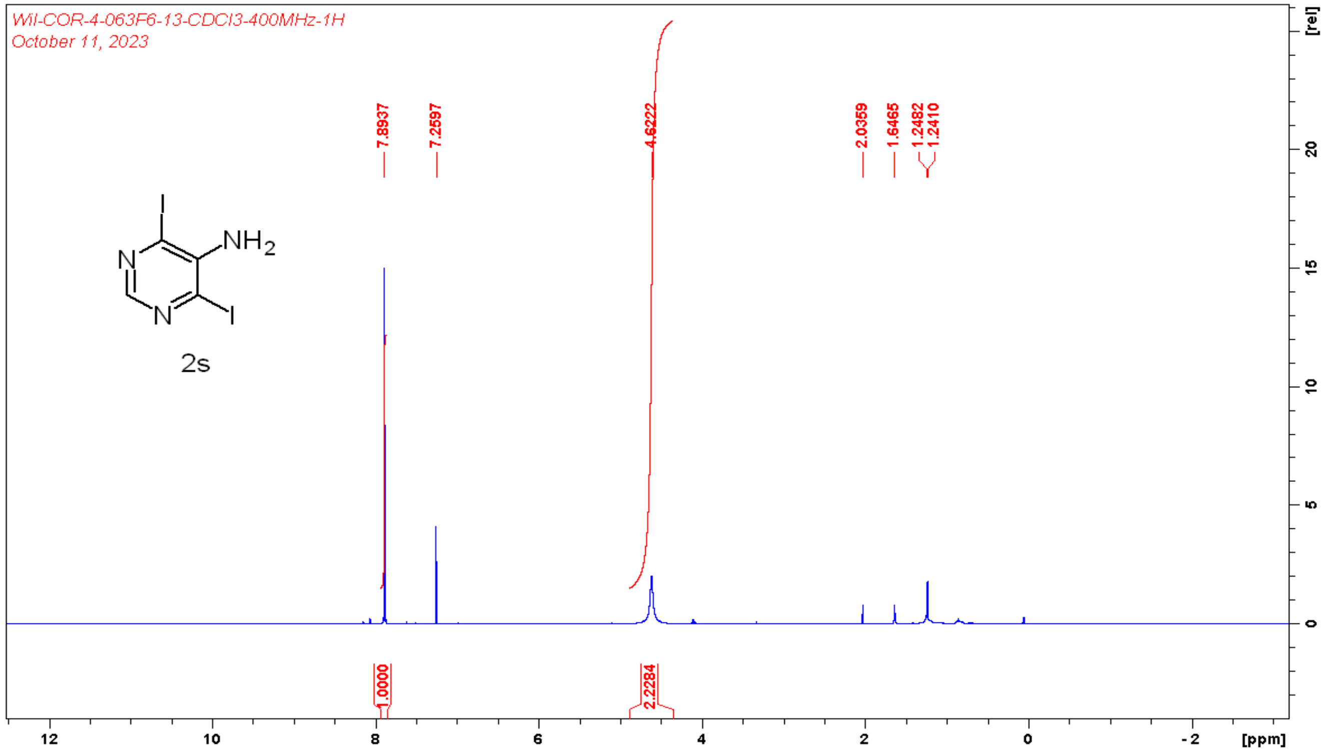

Figure S35: ^{13}C-NMR of 2s in $CDCl_3$

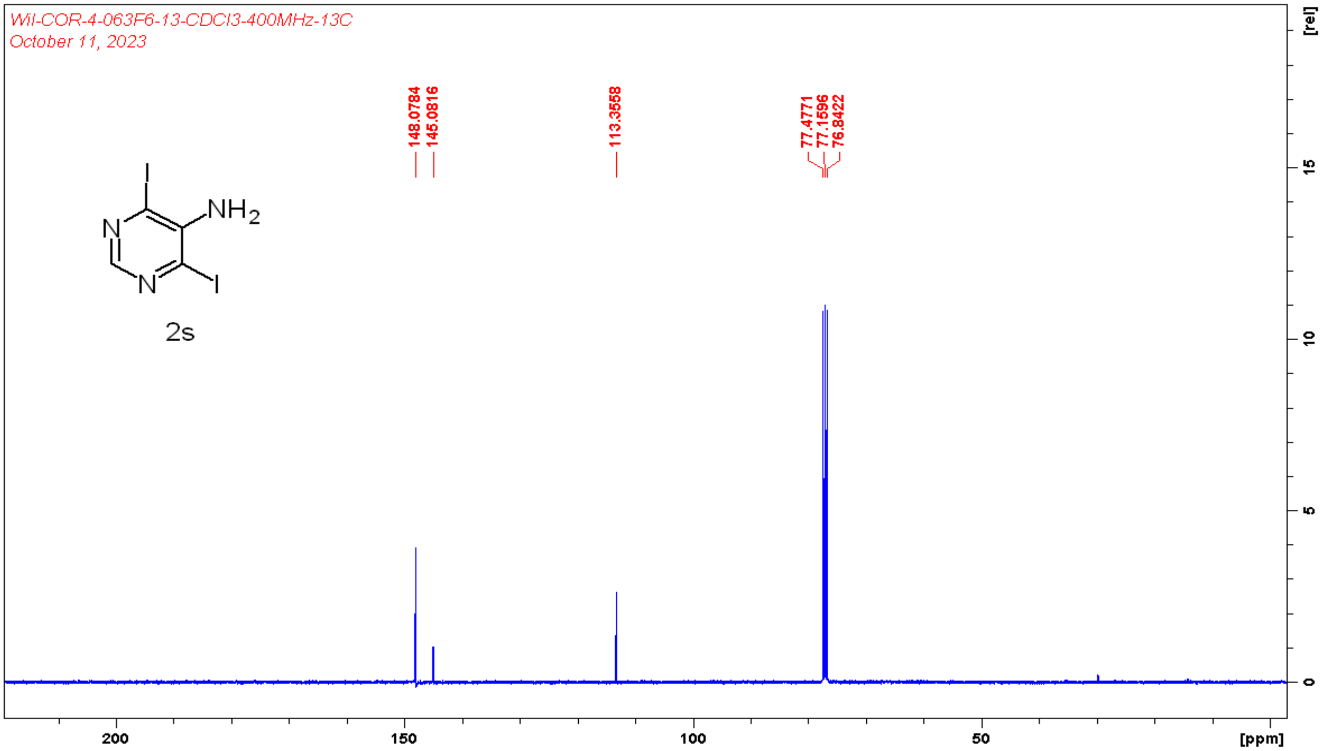

2. References

1. Bi, X.; Li, J.; Shi, E.; Li, Y.; Liu, Y.; Wang, H.; Xiao, J. $POCl_3$ promoted metal-free synthesis of tertiary amides by coupling of carboxylic acids and N,N-disubstituted formamides. *Phosphorus, Sulfur, and Silicon and the Related Elements* **2019**, *194* (3), 236-240.
2. Singh, P.; Choli, A.; Swain, B.; Angeli, A.; Sahoo, S. K.; Yaddanapudi, V. M.; Supuran, C. T.; Arifuddin, M. Design and development of novel series of indole-3-sulfonamide ureido derivatives as selective carbonic anhydrase II inhibitors. *Archiv. der Pharmazie.* **2022**, *355* (1), 2100333.
3. MacNair, A. J.; Tran, M.-M.; Nelson, J. E.; Sloan, G. U.; Ironmonger, A.; Thomas, S. P. Iron-catalysed, general and operationally simple formal hydrogenation using Fe(OTf)3 and NaBH4. *Org. Biomol. Chem.* **2014**, *12* (28), 5082-5088.
4. Grant, D. J.; Dixon, D. A. Heats of Formation and Bond Energies of the H(3−n)BXn Compounds for (X = F, Cl, Br, I, NH_2, OH, and SH). *J. Phys. Chem. A* **2009**, *113* (4), 777-787.
5. Finch, A.; Gardner, P. J.; Hyams, I. J. Thermochemistry of boron tri-iodide and hypoboric acid. *Trans. Faraday Soc.* **1965**, *61* (0), 649-654.
6. Pehlivan, L.; Métay, E.; Laval, S.; Dayoub, W.; Demonchaux, P.; Mignani, G.; Lemaire, M. Alternative method for the reduction of aromatic nitro to amine using TMDS-iron catalyst system. *Tetrahedron* **2011**, *67* (10), 1971-1976.
7. Yang, Z.; Sun, F.; Xu, J.; Li, Y.; Yang, K.; Du, Y., Preparation, Structural Identification and Reactivities of Two Benziodazole-type I(III) Reagents. *Adv. Synth. Catal.* **2023,** *365* (16), 2730-2736.
8. Wang, D.; Kuang, D.; Zhang, F.; Yang, C.; Zhu, X. Room-Temperature Copper-Catalyzed Arylation of Dimethylamine and Methylamine in Neat Water. *Adv. Synth. Catal.* **2015**, *357* (4), 714-718.
9. Jang, M.; Lim, T.; Park, B. Y.; Han, M. S. Metal-Free, Rapid, and Highly Chemoselective Reduction of Aromatic Nitro Compounds at Room Temperature. *J. Org. Chem.* **2022**, *87* (2), 910-919.
10. Cheung, C. W.; Surry, D. S.; Buchwald, S. L. Mild and Highly Selective Palladium-Catalyzed Monoarylation of Ammonia Enabled by the Use of Bulky Biarylphosphine Ligands and Palladacycle Precatalysts. *Org. Lett.* **2013**, *15* (14), 3734-3737.
11. Dubbaka, S. R.; Narreddula, V. R.; Gadde, S.; Mathew, T. Silver-mediated fluorination of potassium aryltrifluoroborates with Selectfluor®. *Tetrahedron* **2014**, *70* (51), 9676-9681.
12. Natte, K.; Jagadeesh, R. V.; He, L.; Rabeah, J.; Chen, J.; Taeschler, C.; Ellinger, S.; Zaragoza, F.; Neumann, H.; Brückner, A.; et al. Palladium-Catalyzed Trifluoromethylation of (Hetero)Arenes with CF_3Br. *Angew. Chem. Int. Ed.* **2016**, *55* (8), 2782-2786.
13. McConnell, N.; Frett, B.; Li, H. Microwave-assisted green synthesis of anilines, phenols, and benzenediamines without transition metals, ligands, or organic solvents. *Green Chem. Lett. Rev.* **2018**, *11* (3), 286-295.
14. Hong, S. Y.; Radosevich, A. T. Chemoselective Primary Amination of Aryl Boronic Acids by PIII/PV═O-Catalysis: Synthetic Capture of the Transient Nef Intermediate HNO. *J. Am. Chem. Soc.* **2022**, *144* (20), 8902-8907.
15. Xue, B.; Shen, J.; Manna, S.; Doppiu, A.; Gooßen, L. J. Selective Monoarylation of Ammonium Triflate with Aryl Chlorides Catalyzed by [Pd(β-MeNAP)Br]2 and AdBrettPhos. *Adv. Synth. Catal.* **2023**, *365* (20), 3473-3477.
16. Kumar, S.; Maurya, S. K. Heterogeneous V2O5/TiO2-Mediated Photocatalytic Reduction of Nitro Compounds to the Corresponding Amines under Visible Light. *J. Org. Chem.* **2023**, *88* (13), 8690-8702.
17. Deng, J.; Cai, Y.; Chen, J.; Wang, Q.; Lu, J.; Chen, X.; Zhang, W.; Wu, K.; Wang, W.; Wei, L. Towards Automated Microfluidic-based Platforms: Optimizing Hydrogenation Efficiency of

Nitrobenzene through π–π Interactions in Pd Nanoparticles on Covalent Organic Frameworks. *Angew. Chem.* **2023**, *135* (23), e202302297.

18. Ibrahim, N.; Legraverend, M. Synthesis of 6,7,8-Trisubstituted Purines via a Copper-Catalyzed Amidation Reaction. *J. Org. Chem.* **2009**, *74* (1), 463-465.
